# Aucubin Ameliorates Alloxan-Induced Diabetic Liver Injury in Association with Modulation of the Nrf2/HO-1 Antioxidant Axis and NF-κB-Associated Inflammatory and Apoptotic Signaling

**DOI:** 10.3390/ijms27188184

**Published:** 2026-09-14

**Authors:** Amany M. Hamed, Nadia S. Mahrous, Safaa S. Soliman, Lobna A. Ali, Rasha Abdeen Refaei, Olivia N. Beshay, Ahmed S. Osman, Marwan Elsayed Eldeeb Mehana Hamouda, Safaa Mohammed Elmahdy, Samira Mahmoud Mohamed, Zeyad Elsayed Eldeeb Mohana, Mohamed S. A. Gaballah, Elsayed Eldeeb Mehana Hamouda, Aboubakr H. Abdelmonsef, Asmaa A. Hegazy

**Affiliations:** 1 Chemistry Department, Faculty of Science, Sohag University, Sohag 82524, Egypt; 2 Department of Zoology, Faculty of Science, Qena University, Qena 83523, Egypt; 3 Department of Zoology, Faculty of Science, Minia University, Minia 61519, Egypt; 4 Cell Biology and Histochemistry, Zoology Department, Faculty of Science, Qena University, Qena 83523, Egypt; 5 Department of Physiology, Faculty of Medicine, Sohag University, Sohag 82524, Egypt; 6 Department of Biochemistry, Faculty of Pharmacy, Minia University, Minia 61519, Egypt; olivia.nady@mu.edu.eg; 7 Department of Biochemistry, Faculty of Pharmacy, Minia National University, New Minia 61768, Egypt; 8 Department of Biochemistry, Faculty of Veterinary Medicine, Sohag University, Sohag 82524, Egypt; 9 College of Medicine, Alexandria University, Alexandria 26571, Egypt; marawanelsayed600@gmail.com (M.E.E.M.H.); zeyadhamouda@gmail.com (Z.E.E.M.); 10 Department of Anatomy, Faculty of Medicine, Sohag University, Sohag 82524, Egypt; 11 Department of Histology and Cell Biology, Faculty of Medicine, Sohag University, Sohag 82524, Egypt; 12 Department of Histology and Cell Biology, Faculty of Medicine, Merit University, Sohag 82524, Egypt; 13 Department of Biochemistry and Molecular Biology, Faculty of Pharmacy, Capital University (Formerly Helwan University), Cairo 11795, Egypt; mohamed.gaballah@pharm.helwan.edu.eg; 14 Department of Pathology, Faculty of Veterinary Medicine, Alexandria University, Alexandria 26571, Egypt; elsayedhamouda@gmail.com; 15 Chemistry Department, Faculty of Science, Qena University, Qena 83523, Egypt; 16 Department of Pathology, Faculty of Veterinary Medicine, Damanhur University, Damanhur 22511, Egypt

**Keywords:** aucubin, diabetes mellitus, hepatoprotection, inflammation, Nrf2/HO-1 pathway, apoptosis

## Abstract

Diabetes mellitus is associated with progressive hepatic injury driven by oxidative stress, inflammation, and apoptosis. Aucubin, a natural iridoid glycoside, possesses potent antioxidant and anti-inflammatory activities; however, its hepatoprotective mechanisms in diabetic liver injury remain unclear. This study investigated the protective effects of aucubin against alloxan-induced diabetic hepatic injury and the underlying molecular mechanisms. Male albino rats were assigned to five groups: normal control, alloxan-induced diabetic, diabetic treated with metformin (150 mg/kg), and diabetic treated with aucubin (25 or 50 mg/kg) for 28 days. We evaluated body weight, fasting blood glucose, liver function, lipid profile, oxidative stress biomarkers, inflammatory cytokines, and hepatic expression of Nrf2, HO-1, NF-κB p65, Bax, and Bcl-2, along with histopathological and immunohistochemical examinations. Alloxan induced marked hyperglycemia, weight loss, hepatic dysfunction, dyslipidemia, oxidative stress, inflammation, and apoptosis. Aucubin significantly ameliorated these alterations, with the 50 mg/kg dose generally showing greater effects than the 25 mg/kg dose. Aucubin improved liver function, ameliorated dyslipidemia, reduced lipid peroxidation, enhanced antioxidant defenses, increased Nrf2 and HO-1 expression, attenuated NF-κB p65 expression and pro-inflammatory cytokines, favorably modulated the Bax/Bcl-2 balance, preserved hepatic architecture, and increased Ki-67 immunoreactivity, indicating enhanced cellular proliferative activity. These findings indicate that aucubin is associated with improved hepatic antioxidant, inflammatory, apoptotic, and metabolic status in alloxan-induced diabetic rats.

## 1. Introduction

Diabetes mellitus (DM) is a chronic metabolic disorder characterized by persistent hyperglycemia resulting from impaired insulin secretion, insulin action, or both. The global prevalence of diabetes has increased dramatically over recent decades, making it one of the leading causes of morbidity and mortality worldwide [1,2]. Beyond disturbances in glucose metabolism, diabetes is recognized as a systemic disease that affects multiple organs, including the liver, kidneys, cardiovascular system, and nervous system. The liver plays a pivotal role in maintaining glucose and lipid homeostasis; therefore, hepatic dysfunction is a major complication of diabetes. Chronic hyperglycemia promotes excessive production of reactive oxygen species (ROS), mitochondrial dysfunction, inflammatory responses, and programmed cell death, all of which contribute to progressive hepatic injury and metabolic impairment [3,4,5].

Experimental animal models remain indispensable for elucidating the molecular mechanisms underlying diabetic complications and for evaluating novel therapeutic agents. Among these models, alloxan-induced diabetes is one of the most extensively employed due to its ability to selectively destroy pancreatic β-cells through ROS generation, resulting in insulin deficiency and sustained hyperglycemia [6]. Although pancreatic β-cell destruction represents the primary target of alloxan, accumulating evidence indicates that its deleterious effects extend beyond the pancreas. The diabetic state induced by alloxan is accompanied by profound hepatic alterations, including hepatocellular degeneration, oxidative damage, inflammatory cell infiltration, lipid metabolic disturbances, and elevated serum liver enzymes, closely resembling hepatic complications observed in diabetic patients [7,8,9].

Oxidative stress is considered a central mechanism in the pathogenesis of diabetic liver injury. Persistent hyperglycemia accelerates glucose auto-oxidation, protein glycation, mitochondrial electron leakage, and activation of NADPH oxidase, collectively leading to excessive ROS production. The resulting oxidative burden overwhelms endogenous antioxidant defenses, causing lipid peroxidation, protein oxidation, DNA damage, and disruption of cellular integrity [10,11]. Consequently, restoration of cellular redox balance has emerged as an attractive therapeutic strategy for preventing diabetes-associated hepatic damage.

Among the endogenous antioxidant defense systems, the nuclear factor erythroid 2-related factor 2 (Nrf2) signaling pathway has attracted considerable attention because of its essential role in maintaining cellular redox homeostasis. Under physiological conditions, Nrf2 remains sequestered in the cytoplasm by Kelch-like ECH-associated protein-1 (Keap1) and undergoes rapid proteasomal degradation. During oxidative stress, Nrf2 dissociates from Keap1 and translocates into the nucleus, where it binds to antioxidant response elements (ARE) and induces the transcription of numerous cytoprotective genes, including heme oxygenase-1 (HO-1), NAD(P)H quinone oxidoreductase-1 (NQO1), glutamate-cysteine ligase, and other phase II detoxifying enzymes [12,13,14,15]. Activation of the Nrf2/HO-1 axis has therefore emerged as a fundamental cellular defense mechanism against oxidative injury in diabetic complications.

In parallel with oxidative stress, chronic inflammation substantially contributes to the progression of diabetic hepatic injury. Nuclear factor kappa B (NF-κB) is regarded as one of the principal transcription factors orchestrating inflammatory responses. Excessive ROS generation activates NF-κB signaling, leading to increased transcription of numerous pro-inflammatory mediators, including tumor necrosis factor-alpha (TNF-α), interleukin-1β (IL-1β), interleukin-6 (IL-6), inducible nitric oxide synthase (iNOS), and cyclooxygenase-2 (COX-2), thereby amplifying hepatic inflammation and tissue injury [16,17,18]. Notably, increasing evidence suggests an intricate reciprocal relationship between Nrf2 and NF-κB signaling pathways, whereby activation of Nrf2 suppresses inflammatory cascades, while persistent NF-κB activation further aggravates oxidative stress, establishing a vicious cycle that accelerates hepatic damage [17,19].

Apoptosis represents another hallmark of diabetes-induced liver injury. Oxidative stress and inflammatory mediators initiate mitochondrial dysfunction, cytochrome c release, and activation of the intrinsic apoptotic pathway by modulating Bcl-2 family proteins and activating caspases. Increased Bax expression together with reduced Bcl-2 levels promotes mitochondrial membrane permeabilization and subsequent activation of caspase-9 and caspase-3, culminating in hepatocyte apoptosis and deterioration of liver architecture [20,21]. Therefore, therapeutic interventions capable of simultaneously attenuating oxidative stress, suppressing inflammation, and preventing apoptosis may provide superior hepatoprotective efficacy.

Natural products have attracted growing interest as potential therapeutic agents for diabetic complications owing to their multitarget pharmacological properties and favorable safety profiles. Aucubin, an iridoid glycoside widely distributed in several medicinal plants such as *Eucommia ulmoides*, *Plantago asiatica*, and *Aucuba japonica*, possesses diverse biological activities, including antioxidant, anti-inflammatory, anti-apoptotic, anti-fibrotic, and cytoprotective effects [22,23]. Previous investigations have demonstrated that aucubin alleviates tissue injury in various experimental models through modulation of oxidative stress and inflammatory signaling pathways. Several studies suggest that activation of Nrf2 signaling, together with inhibition of NF-κB-mediated inflammation, constitutes one of the principal mechanisms underlying its pharmacological actions [24,25,26].

Although several studies have reported the antioxidant, anti-inflammatory, and antidiabetic properties of aucubin in different experimental models, comprehensive mechanistic evidence explaining its hepatoprotective actions during diabetes remains limited. In particular, the coordinated regulation of oxidative stress, inflammatory signaling, apoptosis, and hepatocellular regeneration has not been comprehensively investigated in alloxan-induced diabetic liver injury. Therefore, the present study was designed to investigate the hepatoprotective effects of aucubin against alloxan-induced hepatic injury in diabetic rats through an integrated evaluation of liver function, glycemic status, lipid metabolism, oxidative stress, inflammation, apoptosis, and hepatocellular regeneration using biochemical, molecular, histopathological, and immunohistochemical approaches. Particular emphasis was placed on the Nrf2/HO-1 antioxidant pathway, NF-κB-mediated inflammatory signaling, and Bax/Bcl-2-dependent apoptotic regulation.

## 2. Results

### 2.1. Effect of Aucubin on Body Weight Changes in Alloxan-Induced Diabetic Rats

**Figure 1:**
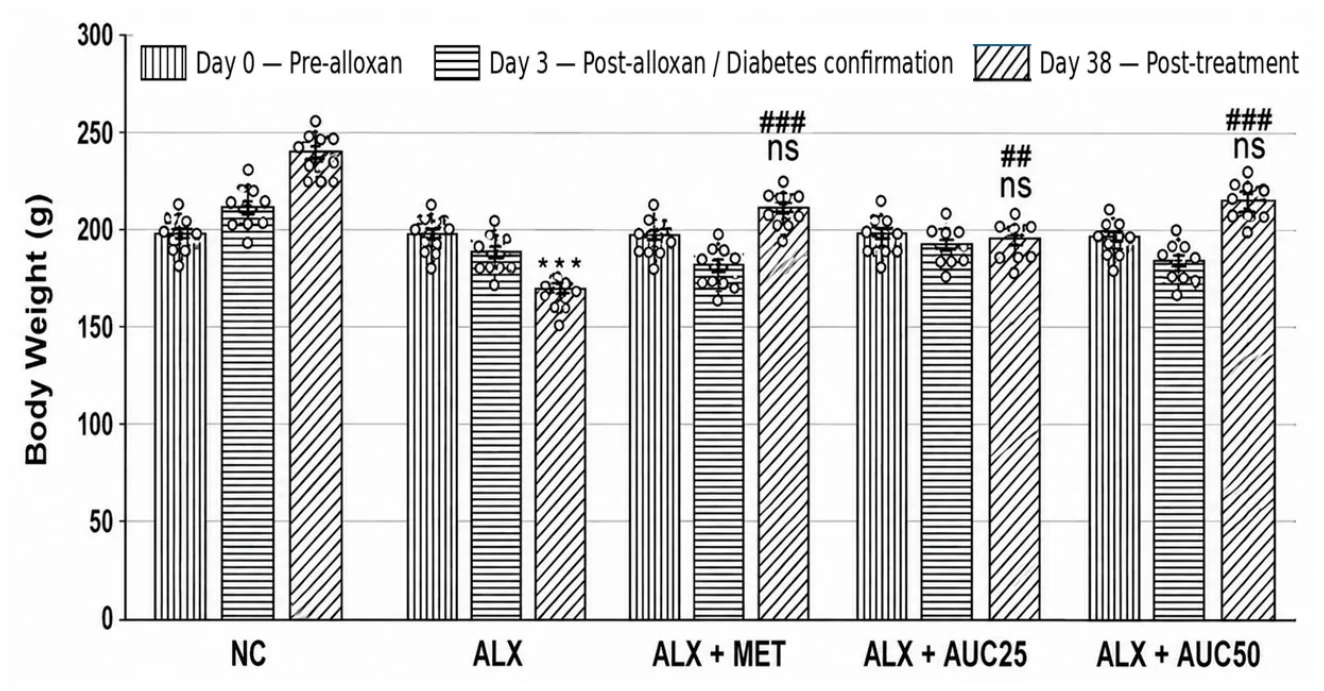
Effect of aucubin treatment on body weight in alloxan-induced diabetic rats. Body weight was assessed at three experimental time points: Day 0—Pre-alloxan (vertically hatched bars), Day 3—Post-alloxan/Diabetes confirmation (horizontally hatched bars), and Day 38—Post-treatment (diagonally hatched bars). Data are presented as the mean ± SD (*n* = 8), with individual data points shown. We performed repeated-measures ANOVA, with time as the within-subject factor and experimental group as the between-subject factor, followed by Tukey’s multiple comparison test. We also assessed the group × time interaction. ns, not significant; *** *p* < 0.001 versus the NC group; ^##^ *p* < 0.01, and ^###^ *p* < 0.001 versus the alloxan-induced diabetic (ALX) group. NC, normal control; ALX, alloxan-induced diabetic group; MET, metformin (reference antidiabetic drug); AUC25, aucubin (25 mg/kg); AUC50, aucubin (50 mg/kg).

Figure 1 shows no significant differences in body weight among the experimental groups at Day 0 (Pre-alloxan), indicating comparable initial body weights. At Day 3 (Post-alloxan/Diabetes confirmation), the ALX group exhibited a significant reduction in body weight compared with the NC group (*p* < 0.001), consistent with the development of the diabetic phenotype.

At Day 38 (Post-treatment), the ALX group exhibited a further decline in body weight compared with the NC group (*p* < 0.001). In contrast, metformin treatment (ALX + MET) significantly attenuated body weight loss compared with the untreated ALX group (*p* < 0.001), with body weight showing no significant difference from the NC group. Similarly, aucubin treatment significantly improved body weight compared with the untreated ALX group. AUC25 significantly attenuated body weight loss compared with the ALX group (*p* < 0.01), whereas AUC50 produced a more pronounced recovery of body weight compared with the untreated ALX group. Overall, these findings indicate that aucubin ameliorated diabetes-associated body weight loss, with the higher dose exhibiting greater efficacy.

### 2.2. Effect of Aucubin on Fasting Blood Glucose Levels

**Figure 2:**
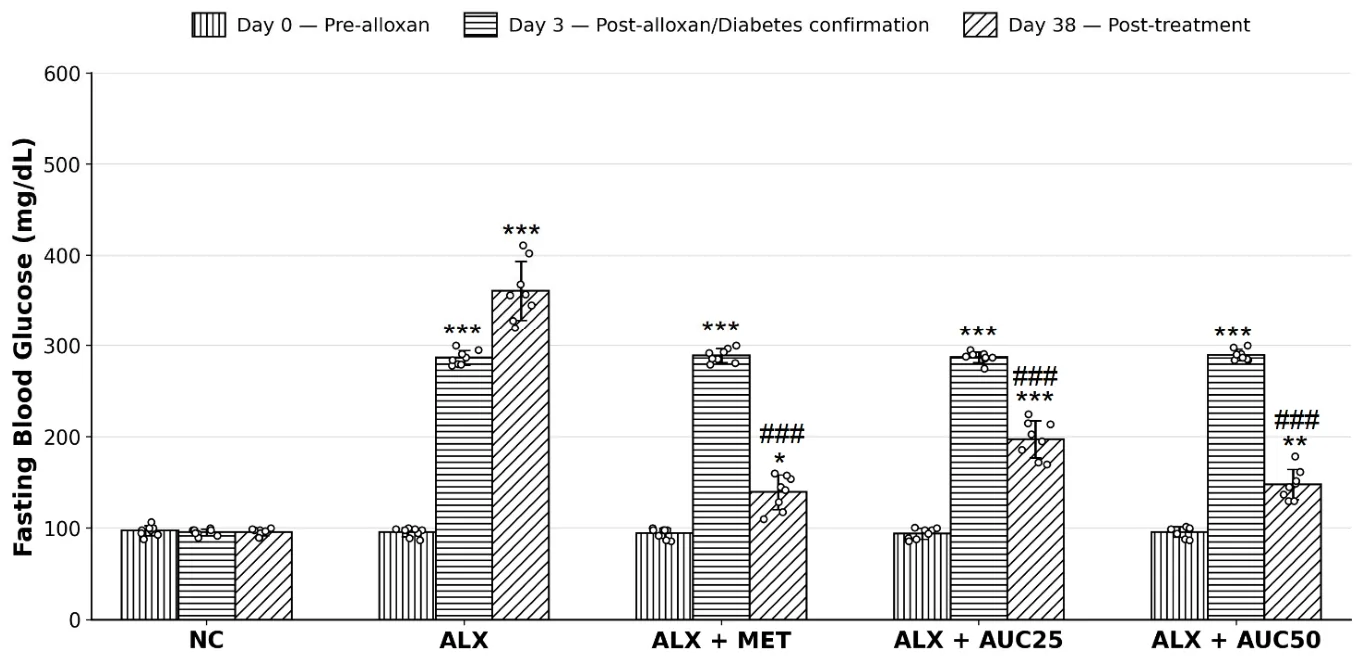
Effect of aucubin on fasting blood glucose levels in alloxan-induced diabetic rats. Fasting blood glucose (FBG) was measured at Day 0 (Pre-alloxan), Day 3 (Post-alloxan/diabetes confirmation; 72 h after alloxan administration), and Day 38 (Post-treatment; at the end of the 28-day treatment period). Data are expressed as the mean ± SD (*n* = 8), with individual data points shown. We performed repeated-measures ANOVA, with time as the within-subject factor and experimental group as the between-subject factor, followed by Tukey’s multiple comparison test. The group × time interaction was also assessed. * *p* < 0.05, ** *p* < 0.01, and *** *p* < 0.001 versus the normal control (NC) group; ^###^ *p* < 0.001 versus the alloxan-induced diabetic (ALX) group. NC, normal control; ALX, alloxan-induced diabetic group; MET, metformin (reference antidiabetic drug); AUC25, aucubin (25 mg/kg); AUC50, aucubin (50 mg/kg).

As shown in Figure 2, fasting blood glucose (FBG) levels were comparable among all experimental groups before diabetes induction, with no significant differences observed. Seventy-two hours after alloxan (ALX) administration, diabetic rats exhibited a marked elevation in FBG levels compared with the normal control (NC) group (*p* < 0.001), confirming the successful induction of experimental diabetes.

At the end of the treatment period, untreated ALX rats exhibited persistently elevated FBG levels compared with the NC group (*p* < 0.001). In contrast, metformin treatment significantly reduced FBG levels compared with the ALX group (*p* < 0.001), although glucose levels remained significantly higher than those of the NC group (*p* < 0.05).

Similarly, aucubin administration produced a significant antihyperglycemic effect. Treatment with aucubin at 25 mg/kg significantly decreased FBG levels compared with untreated diabetic rats (*p* < 0.001); however, glucose levels remained significantly higher than those of the NC group (*p* < 0.001). Likewise, treatment with aucubin at 50 mg/kg significantly reduced FBG levels relative to the ALX group (*p* < 0.001), although FBG levels remained significantly higher than those of the NC group (*p* < 0.01).

Collectively, these findings demonstrate that aucubin effectively attenuated alloxan-induced hyperglycemia, with a greater reduction in FBG observed at 50 mg/kg than at 25 mg/kg.

### 2.3. Effect of Aucubin on Liver Function Biomarkers

As presented in Table 1, alloxan-induced diabetes caused marked hepatic dysfunction, as evidenced by significant elevations in serum AST, ALT, and ALP activities, accompanied by a significant reduction in serum albumin levels compared with the normal control (NC) group (*p* < 0.001).

Treatment with metformin significantly ameliorated liver injury by reducing serum AST and ALT levels (*p* < 0.01 vs. NC; *p* < 0.01 vs. ALX) and ALP activity to a level comparable with the NC group (ns vs. NC; *p* < 0.001 vs. ALX). In addition, metformin significantly increased serum albumin compared with the untreated diabetic group (*p* < 0.01 vs. NC; *p* < 0.01 vs. ALX), although albumin remained significantly lower than in the NC group.

Similarly, aucubin treatment markedly improved liver function biomarkers in diabetic rats. Administration of AUC25 (25 mg/kg) significantly decreased AST, ALT, and ALP activities compared with the ALX group (*p* < 0.001), and these parameters remained significantly different from those of the NC group (*p < 0.05*). Moreover, serum albumin increased significantly after AUC25 treatment compared with the ALX group (*p* < 0.001), although it did not fully return to normal levels (*p* < 0.01 vs. NC).

Treatment with AUC50 (50 mg/kg) produced a more pronounced hepatoprotective effect, restoring serum AST, ALT, and ALP activities to levels that were not significantly different from those of the NC group (ns), while remaining significantly improved compared with the ALX group (*p* < 0.001). Likewise, serum albumin was markedly increased relative to the untreated diabetic group (*p* < 0.001) and reached a level comparable with that of the NC group (ns). Collectively, these findings demonstrate that aucubin effectively attenuated alloxan-induced hepatic dysfunction, with the 50 mg/kg dose showing greater overall improvement than the 25 mg/kg dose.

### 2.4. Effect of Aucubin on Serum Lipid Profile

As shown in Table 2, alloxan-induced diabetes resulted in marked dyslipidemia, as evidenced by significant increases in serum total cholesterol (CH), triglycerides (TG), and low-density lipoprotein cholesterol (LDL) levels, accompanied by a significant reduction in high-density lipoprotein cholesterol (HDL) compared with the normal control (NC) group (*p* < 0.001).

Treatment with metformin significantly improved the lipid profile of diabetic rats. Serum CH, TG, HDL, and LDL levels were significantly ameliorated compared with the untreated diabetic (ALX) group (*p* < 0.01); however, these parameters remained significantly different from those of the NC group (*p* < 0.01).

Similarly, administration of aucubin at 25 mg/kg (AUC25) significantly attenuated diabetes-induced dyslipidemia. Compared with the ALX group, AUC25 significantly reduced serum CH, TG, and LDL levels while significantly increasing HDL concentrations (*p* < 0.01). Nevertheless, all lipid parameters remained significantly different from those observed in the NC group (*p* < 0.05).

Treatment with aucubin at 50 mg/kg (AUC50) produced a more pronounced improvement in lipid homeostasis. Serum TC, TG, HDL-C, and LDL-C levels were significantly improved compared with the ALX group (*p* < 0.001) and showed no significant differences from those of the NC group. Collectively, these findings indicate that aucubin effectively alleviates alloxan-induced dyslipidemia, with the 50 mg/kg dose demonstrating superior lipid-lowering efficacy compared with the 25 mg/kg dose, restoring the lipid profile to near-normal levels.

### 2.5. Effect of Aucubin on Hepatic Oxidative Stress Biomarkers

As presented in Table 3, alloxan-induced diabetes markedly disrupted hepatic redox homeostasis, as evidenced by a significant increase in hepatic malondialdehyde (MDA) levels, accompanied by significant reductions in reduced glutathione (GSH) content and the antioxidant enzyme activities of superoxide dismutase (SOD) and catalase (CAT) compared with the normal control (NC) group (*p* < 0.001).

Treatment with metformin significantly attenuated oxidative stress, as demonstrated by a reduction in MDA levels and significant increases in GSH content, SOD activity, and CAT activity compared with the untreated diabetic (ALX) group (*p* < 0.01). However, all oxidative stress biomarkers remained significantly different from those of the NC group (*p* < 0.01).

Similarly, administration of aucubin at 25 mg/kg (AUC25) markedly improved hepatic oxidative status. Compared with the ALX group, AUC25 significantly decreased MDA levels while significantly restoring GSH content and SOD and CAT activities (*p* < 0.001). Nevertheless, these parameters remained significantly different from those of the NC group (*p* < 0.05).

Treatment with aucubin at 50 mg/kg (AUC50) produced a more pronounced antioxidant effect. Hepatic MDA levels were markedly reduced, whereas GSH content and the activities of SOD and CAT were significantly increased compared with the ALX group (*p* < 0.001). Importantly, all four oxidative stress biomarkers were no longer significantly different from those of the NC group (ns), indicating near-complete restoration of hepatic redox balance. Collectively, these findings demonstrate that aucubin effectively ameliorated alloxan-induced oxidative stress, with the 50 mg/kg dose showing greater antioxidant efficacy than the 25 mg/kg dose.

### 2.6. Effect of Aucubin on Hepatic Pro-Inflammatory Cytokines

**Figure 3:**
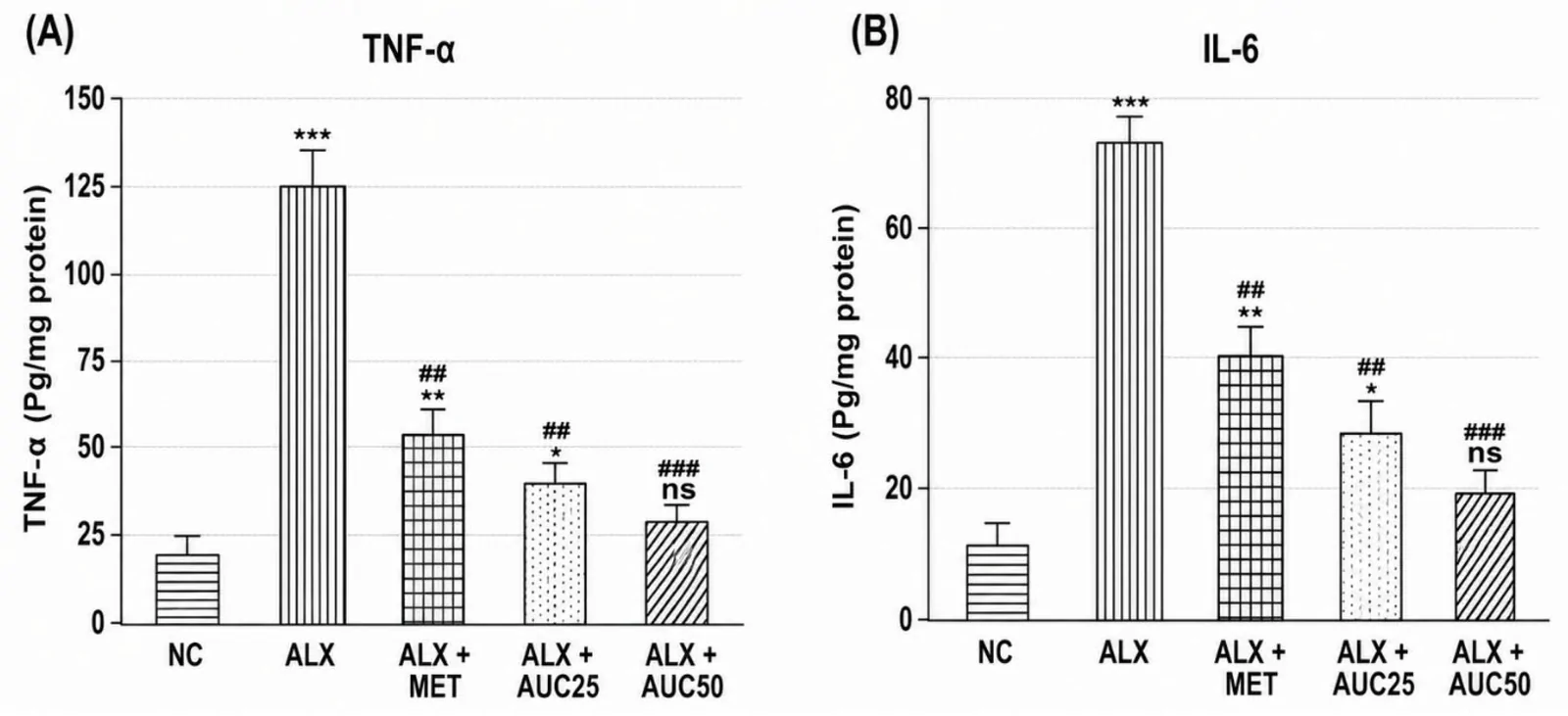
Effect of aucubin on hepatic pro-inflammatory cytokine levels in alloxan-induced diabetic rats. Hepatic levels of (**A**) tumor necrosis factor-alpha (TNF-α) and (**B**) interleukin-6 (IL-6) were determined after 28 days of treatment. Data are expressed as the mean ± SD (*n* = 8 rats per group). Statistical analysis was performed using one-way ANOVA followed by Tukey’s multiple comparison test. *ns*, not significant; * *p* < 0.05, ** *p* < 0.01, and *** *p* < 0.001 versus the normal control (NC) group; ^##^ *p* < 0.01, and ^###^ *p* < 0.001 versus the alloxan-induced diabetic (ALX) group. NC, normal control; ALX, alloxan-induced diabetic group; MET, metformin (reference antidiabetic drug); AUC25, aucubin (25 mg/kg); AUC50, aucubin (50 mg/kg); TNF-α, tumor necrosis factor-alpha; IL-6, interleukin-6.

As shown in Figure 3, alloxan-induced diabetes markedly enhanced hepatic inflammation, as evidenced by significant increases in the hepatic levels of tumor necrosis factor-alpha (TNF-α) and interleukin-6 (IL-6) compared with the normal control (NC) group (*p* < 0.001).

Treatment with metformin significantly attenuated the inflammatory response, resulting in marked reductions in hepatic TNF-α and IL-6 levels compared with the untreated diabetic (ALX) group (*p* < 0.01). However, both cytokines remained significantly elevated relative to the NC group (*p* < 0.01).

Similarly, treatment with aucubin at 25 mg/kg (AUC25) significantly decreased hepatic TNF-α and IL-6 levels compared with the ALX group (*p* < 0.01), although both cytokines remained significantly higher than those of the NC group (*p* < 0.05).

Notably, administration of aucubin at 50 mg/kg (AUC50) produced the greatest anti-inflammatory effect. Hepatic TNF-α and IL-6 levels were significantly reduced compared with the untreated diabetic group (*p* < 0.001) and showed no significant differences from those of the NC group. Collectively, these findings indicate that aucubin effectively attenuated alloxan-induced hepatic inflammation, with the 50 mg/kg dose showing greater anti-inflammatory effects than the 25 mg/kg dose.

### 2.7. Effect of Aucubin on the Expression of Nrf2, HO-1, NF-κB, Bax, and Bcl-2 Proteins

**Figure 4:**
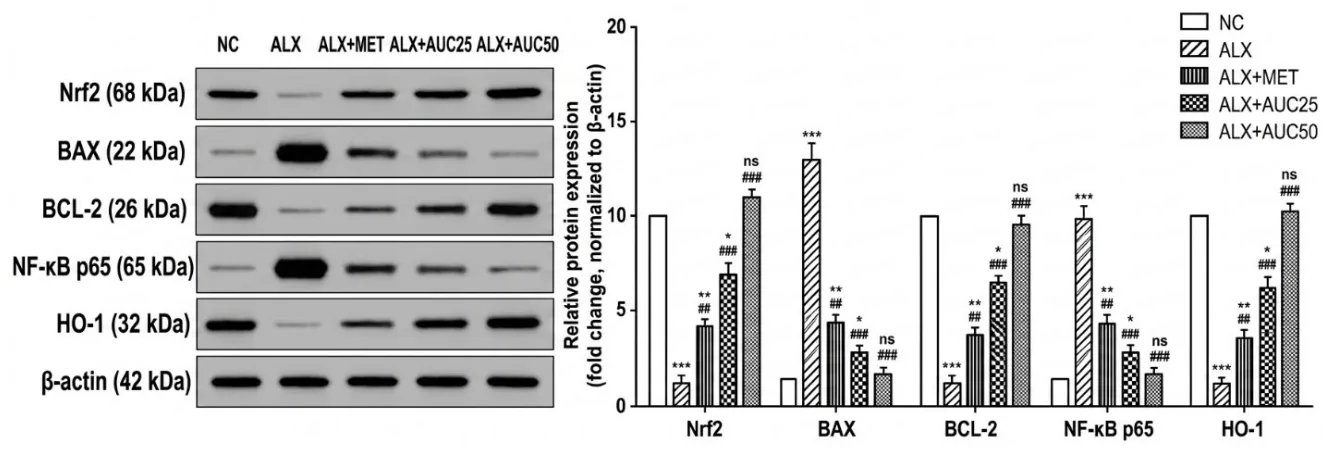
Effect of aucubin on hepatic protein expression of oxidative stress-, inflammatory-, and apoptosis-related markers in alloxan-induced diabetic rats. Representative Western blot images (left) and densitometric analysis (right) of nuclear factor erythroid 2-related factor 2 (Nrf2), heme oxygenase-1 (HO-1), nuclear factor kappa B p65 (NF-κB p65), B-cell lymphoma 2 (BCL-2), and Bcl-2-associated X protein (BAX) in hepatic tissue. Target protein expression was normalized to β-actin and expressed as fold change relative to the normal control (NC) group. Data are presented as mean ± SD (*n* = 8 rats per group). Statistical analysis was performed using one-way ANOVA followed by Tukey’s multiple comparison test. ns, not significant; * *p* < 0.05, ** *p* < 0.01, and *** *p* < 0.001 versus the NC group; ^##^ *p* < 0.01, and ^###^ *p* < 0.001 versus the ALX group. NC, normal control; ALX, alloxan-induced diabetic group; MET, metformin (reference antidiabetic drug); AUC25, aucubin (25 mg/kg); AUC50, aucubin (50 mg/kg).

As illustrated in Figure 4, alloxan-induced diabetes markedly altered the hepatic expression of proteins involved in oxidative stress, inflammation, and apoptosis. Compared with the normal control (NC) group, diabetic rats exhibited significant downregulation of the antioxidant proteins Nrf2 and HO-1, accompanied by marked upregulation of the pro-inflammatory marker NF-κB p65 and the pro-apoptotic protein Bax, together with a pronounced reduction in the anti-apoptotic protein Bcl-2 (*p* < 0.001), indicating the development of substantial oxidative stress, inflammatory activation, and apoptotic signaling in the diabetic liver.

Treatment with metformin significantly ameliorated these diabetes-associated molecular alterations. Compared with the untreated diabetic (ALX) group, metformin increased the expression of Nrf2, HO-1, and Bcl-2 while reducing the expression of NF-κB p65 and Bax (*p* < 0.01). However, the expression levels of these proteins remained significantly different from those of the NC group, indicating incomplete restoration toward normal values.

Similarly, administration of aucubin at 25 mg/kg (AUC25) significantly modulated hepatic protein expression, as evidenced by increased expression of Nrf2, HO-1, and Bcl-2 and reduced expression of NF-κB p65 and Bax compared with the ALX group (*p* < 0.001). Nevertheless, these protein levels remained significantly different from those of the NC group (*p* < 0.05), indicating partial recovery following AUC25 treatment.

Notably, aucubin at 50 mg/kg (AUC50) produced the most pronounced normalization of the evaluated molecular markers. Compared with the ALX group, AUC50 significantly increased Nrf2, HO-1, and Bcl-2 expression while markedly attenuating the diabetes-associated elevation of NF-κB p65 and Bax (*p* < 0.001). Importantly, none of the evaluated protein expression levels differed significantly from those of the NC group, suggesting substantial restoration of the hepatic molecular profile toward normal conditions.

Collectively, these findings indicate that aucubin attenuated the diabetes-associated alterations in hepatic antioxidant, inflammatory, and apoptotic signaling, as evidenced by restoration of the Nrf2/HO-1 antioxidant axis, suppression of NF-κB p65-associated inflammatory signaling, and normalization of the Bax/Bcl-2 apoptotic balance. The more pronounced molecular normalization observed with AUC50 than with AUC25 indicates a greater effect at the higher dose under the present experimental conditions.

### 2.8. Effect of Aucubin on Hepatic Histopathological Alterations

**Figure 5:**
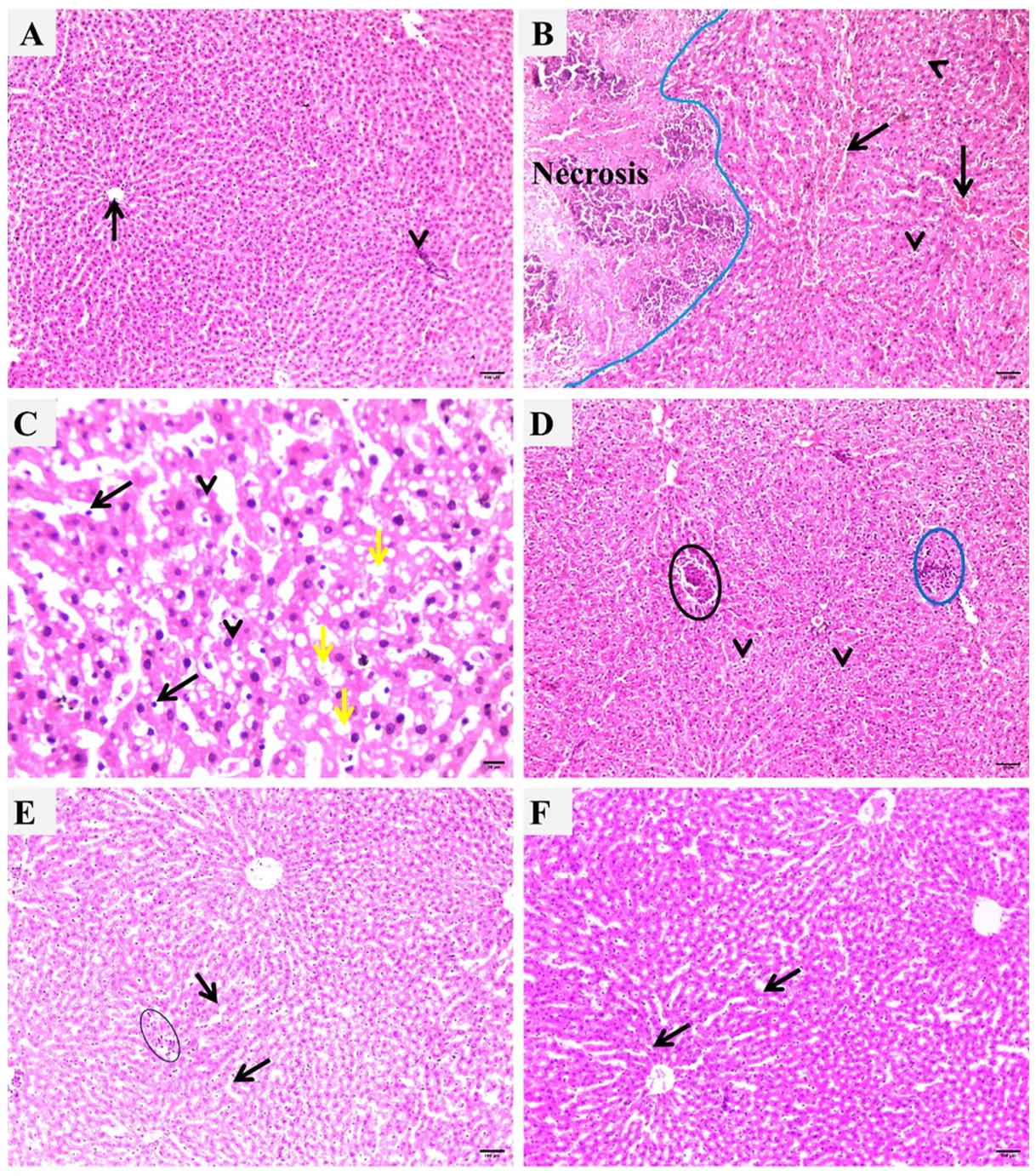
Histological sections of liver tissue from different study groups: (**A**) control group showed normal hepatic morphology with identified central vein (black arrow), portal area (black arrowhead), and uniform hepatic cords, separated by radiating sinusoids. (**B**,**C**) Liver tissue of alloxan-induced diabetic rats showed large areas of necrosis with dense inflammation, congested central veins and sinusoids (black arrows), and vacuolated hepatocytes (black arrowheads). Hepatocytes showed frequent pyknotic nuclei (black arrows), microvesicular (black arrowheads) and macrovesicular (yellow arrows) steatosis; In panel B, the blue line demarcates the boundary between the necrotic area and the relatively preserved hepatic parenchyma. (**D**) Metformin-treated rats showed mild degenerative changes (vacuolar degeneration; black arrowheads), a focal necrotic area (black circle), and a focal area of inflammation (blue circle). (**E**) Aucubin at 25 mg/kg showed restoration of the normal hepatic architecture, with preserved hepatic cords radiating from the central vein. Hepatocytes exhibit only minimal vacuolar degeneration, mild sinusoidal dilatation (black arrows), and a small focal aggregate of mononuclear inflammatory cells (circled area). (**F**) Aucubin at 50 mg/kg induced remarkable improvement of hepatic morphology. Hepatocytes have almost retained their architecture with preserved hepatocyte cording (black arrows) and intervening sinusoids. H&E-stained sections; magnification is ×400 for C and ×100 for others.

Table 4 summarizes histopathologic findings in hepatic tissues for all experimental groups. The liver of the control group displayed a normal hepatic architecture characterized by well-defined hepatic lobules with distinct central veins and portal triads. Hepatocytes were arranged in one-to-two-cell-thick cords radiating from the central vein and separated by intact hepatic sinusoids. The hepatocytes displayed a typical polygonal morphology with eosinophilic cytoplasm and centrally located, uniform nuclei (Figure 5A).

The alloxan-induced diabetes group exhibited significant hepatic tissue damage (Figure 5B,C). Extensive coagulative necrosis was observed, accompanied by disruption of the normal hepatic architecture and intense inflammatory cell infiltration. The remaining hepatic parenchyma showed marked hepatocellular degeneration, characterized by hydropic (cloudy) swelling, cytoplasmic vacuolation, and micro- and macrovesicular steatosis. In addition, marked congestion and dilation of the central veins and hepatic sinusoids were evident.

Metformin treatment had little enhancement in hepatic morphological alterations compared to alloxan-induced diabetic rats. Residual mild venous and sinusoidal congestion, patchy microvesicular steatosis, and hazy swelling of hepatocytes were present. A focal aggregate of mononuclear inflammatory cells was observed within the hepatic parenchyma, admixed with a few activated Kupffer cells (Figure 5D).

Administration of aucubin at 25 mg/kg (AUC25) markedly ameliorated the diabetes-induced histopathological alterations in hepatic architecture. Hepatic cords were regularly arranged around the central vein, and the overall lobular tissue was largely preserved. Mild residual hepatocellular degeneration, characterized by slight cytoplasmic vacuolation, was observed in scattered hepatocytes. Sinusoidal dilatation persisted, together with a small focal aggregate of mononuclear inflammatory cells. Considerably, no areas of coagulative necrosis were detected (Figure 5E).

Notably, treatment with aucubin at 50 mg/kg (AUC50) provided superior hepatoprotective effect, resulting in near-complete restoration of the normal hepatic architecture and marked attenuation of diabetes-induced histopathological alterations. The hepatic lobules were well organized, with hepatocytes arranged in regular cords radiating from the central veins and separated by patent sinusoids. Hepatocytes displayed normal polygonal morphology with centrally located nuclei. No evidence of hepatocellular necrosis, marked sinusoidal congestion, or severe degenerative changes was detected (Figure 5F).

### 2.9. Effect of Aucubin on Hepatic Immunohistochemical Expression of Nrf2, NF-κB, Caspase-3, and Ki67

Semi-quantitative immunohistochemical analysis revealed significant differences among the experimental groups in hepatic NF-κB (p65), Nrf2, cleaved caspase-3, and Ki-67 expression (Table 5).

Expression of Nrf2

**Figure 6:**
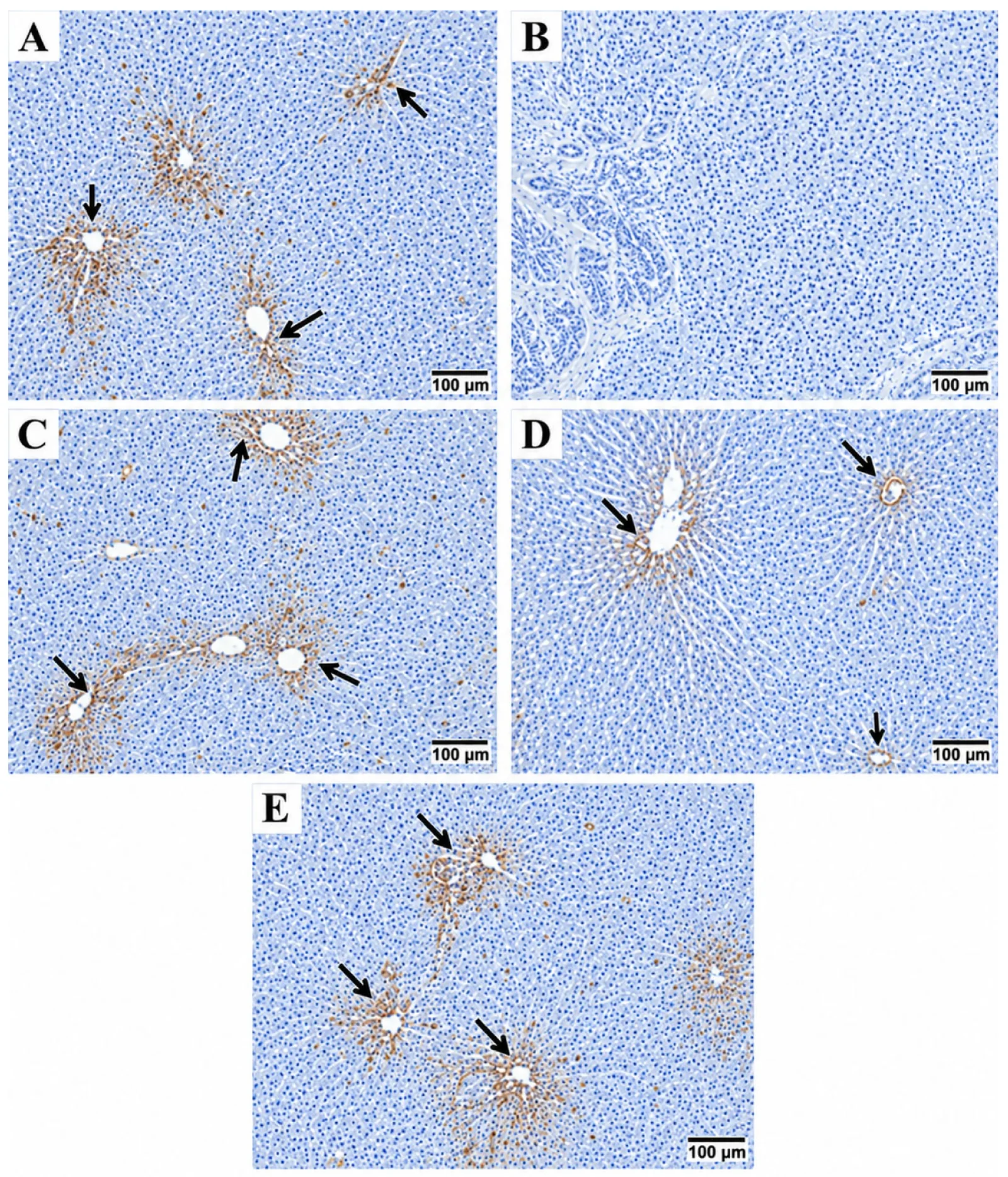
Expression of Nrf2 in hepatic tissue of different study groups: (**A**) Cytoplasmic immune-staining of Nrf2 in hepatocytes from control rats (black arrows). (**B**) Absence of Nrf2 expression throughout the hepatic tissue obtained from alloxan-induced diabetic rats. (**C**) Metformin-treated rats showed mild restoration of Nrf2 immunoreactivity in hepatocytes (black arrows). (**D**) Aucubin 25 mg/kg (AUC25)-treated group exhibited moderate Nrf2 immunoreactivity in hepatocytes (black arrows). (**E**) Aucubin 50 mg/kg (AUC50)-treated group exhibited marked Nrf2 immunoreactivity, approaching the normal staining pattern (black arrows). Immunostained sections; magnification is ×100 for all.

Nrf2 immunoreactivity was detected as brown granular cytoplasmic staining in hepatocytes, with more prominent staining in the pericentral region surrounding the central veins. The NC group exhibited moderate cytoplasmic Nrf2 immunoreactivity, predominantly in pericentral hepatocytes, with an immunoreactivity score (IRS) of 6.50 ± 2.00 (Figure 6A). In contrast, the ALX group showed a marked reduction in Nrf2 staining, with minimal immunoreactivity in most hepatocytes (Figure 6B), resulting in a significantly lower IRS of 0.50 ± 0.76 than that of the NC group (*p* < 0.05). The MET-treated group showed partial restoration of Nrf2 immunoreactivity (Figure 6C), with an IRS of 2.50 ± 1.31, which remained significantly lower than that of the NC group (*p* < 0.05).

A further increase in Nrf2 immunoreactivity was observed following aucubin treatment. The AUC25 group exhibited moderate and more widespread cytoplasmic staining in hepatocytes (Figure 6D), with an IRS of 4.38 ± 2.13. The AUC50 group showed widespread Nrf2-positive hepatocytes, with staining intensity and distribution approaching those observed in the NC group (Figure 6E), and an IRS of 6.38 ± 1.85. The IRS values of both AUC25 and AUC50 were significantly higher than those of the ALX group, while neither differed significantly from the NC group. Overall, these findings indicate that aucubin treatment was associated with restoration of hepatic Nrf2 immunoreactivity in alloxan-induced diabetic rats, with the 50 mg/kg dose producing the greatest restoration.

Expression of NF-κB (p65)

**Figure 7:**
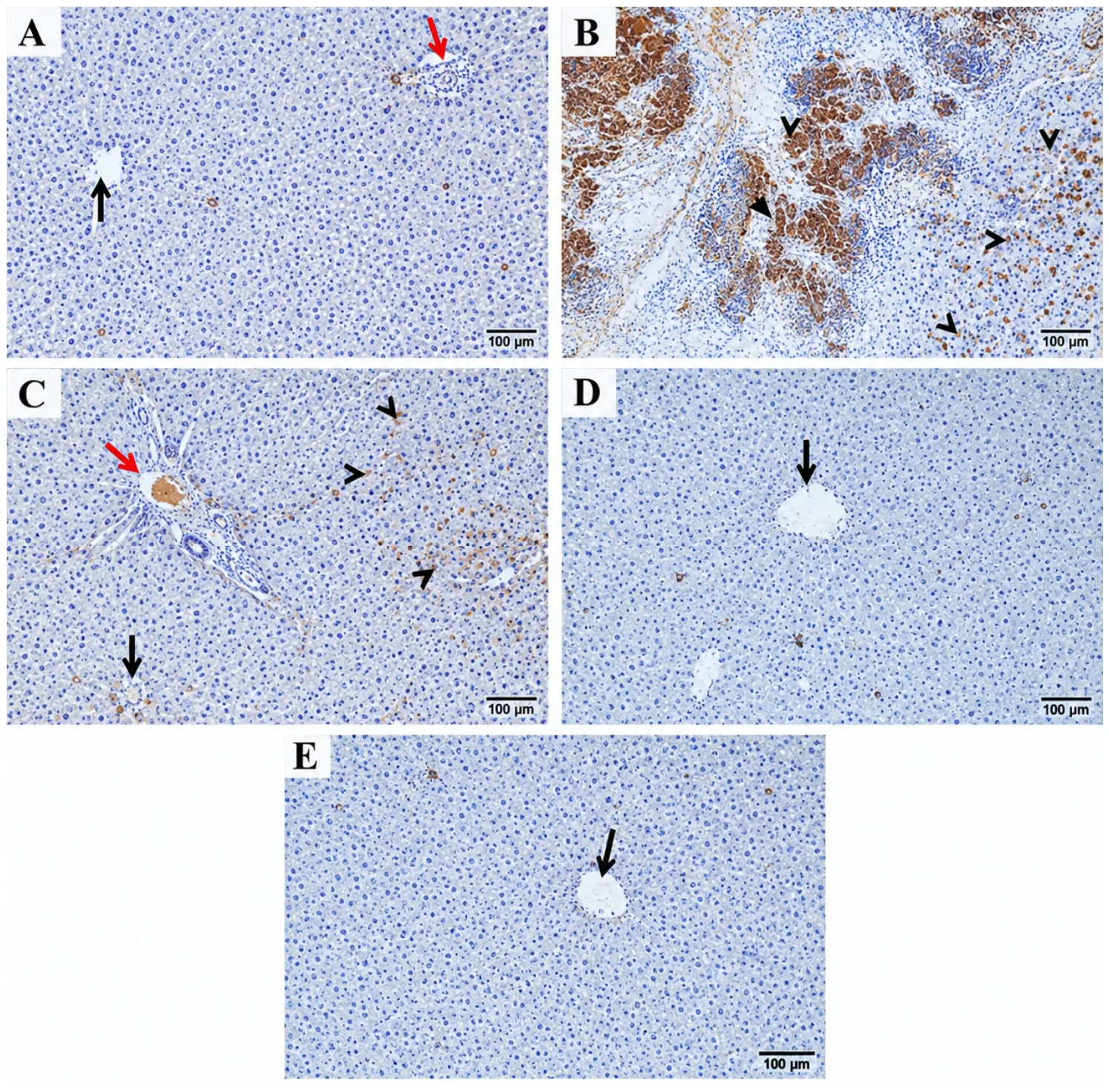
Expression of NF-κB in hepatic tissue of different study groups: (**A**) Negative expression of NF-κB in hepatic tissue from control rats; the central vein (black arrow), portal area (red arrow). (**B**) Intense expression of NF-κB throughout the hepatic tissue obtained from alloxan-induced diabetic rats, in both the necrotic area and other hepatocytes (black arrowheads). (**C**) Metformin-treated rats showed mild NF-κB immunoreactivity in hepatocytes (black arrowheads); the central vein (black arrow), portal area (red arrow). (**D**) Aucubin at 25 mg/kg (AUC25) and (**E**) Aucubin at 50 mg/kg (AUC50) treated groups did not express NF-κB; the central vein (black arrow). Immunostained sections; magnification is ×100 for all.

Immunohistochemical staining for NF-κB (p65), a marker associated with the inflammatory response, was detected as brown cytoplasmic immunoreactivity in hepatocytes and inflammatory cells. The NC group showed minimal NF-κB (p65) immunoreactivity, with a low IRS of 0.50 ± 0.76 (Figure 7A). In contrast, the ALX group exhibited intense NF-κB (p65) immunoreactivity, particularly in necrotic areas associated with inflammatory cell infiltration, as well as in viable hepatocytes (Figure 7B), resulting in a significantly higher IRS of 8.50 ± 2.51 than that of the NC group (*p* < 0.05). Treatment with MET markedly reduced NF-κB (p65) immunoreactivity, with an IRS of 2.00 ± 1.07 (Figure 7C). A further reduction was observed following aucubin treatment, with IRS values of 0.62 ± 0.74 and 0.38 ± 0.52 in the AUC25 and AUC50 groups, respectively (Figure 7D,E). The IRS values of the MET, AUC25, and AUC50 groups were significantly lower than those of the ALX group, while none differed significantly from the NC group.

Expression of Caspase-3

**Figure 8:**
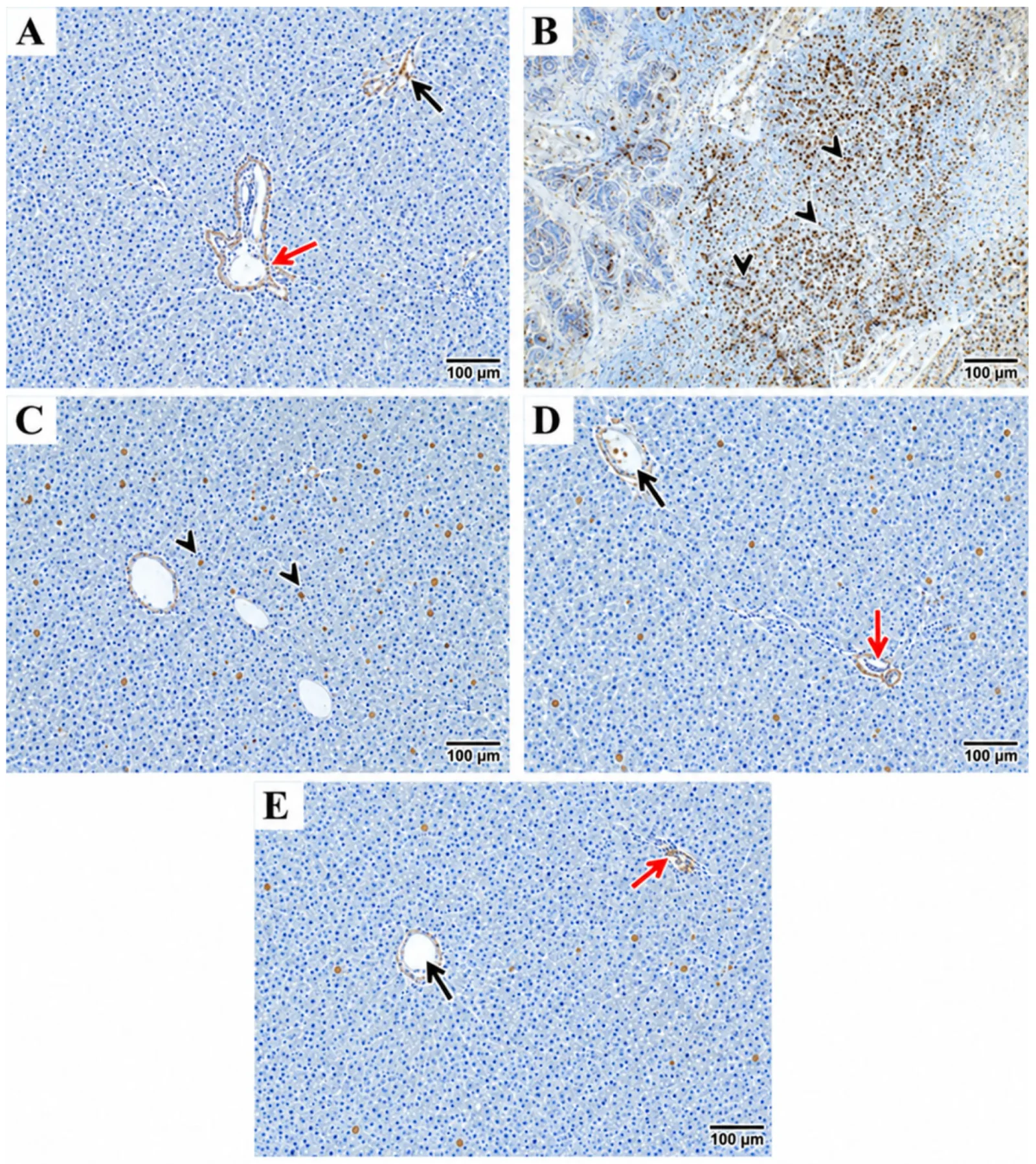
Expression of cleaved caspase-3 in hepatic tissue of different study groups: (**A**) Control group showing negative cleaved caspase-3 immunoreactivity in hepatocytes; the central vein (black arrow), portal area (red arrow). (**B**) Alloxan-induced diabetic group exhibiting marked cleaved caspase-3 expression throughout the hepatic parenchyma (black arrowheads). (**C**) Metformin-treated group showing mild cleaved caspase-3 immunoreactivity in scattered hepatocytes (black arrowheads). (**D**) Aucubin-treated group at 25 mg/kg (AUC25); the central vein (black arrow), portal area (red arrow) and (**E**) 50 mg/kg (AUC50) did not have cleaved caspase-3 expression; the central vein (black arrow), portal area (red arrow). Immunostained sections; magnification is ×100 for all.

Immunohistochemical staining for cleaved caspase-3, an established marker of apoptosis, revealed minimal cytoplasmic immunoreactivity in hepatocytes of the NC group, with a low IRS of 0.62 ± 0.92 (Figure 8A). Conversely, the ALX group exhibited strong cytoplasmic cleaved caspase-3 immunoreactivity in numerous hepatocytes (Figure 8B), resulting in a significantly higher IRS of 9.75 ± 2.66 than that of the NC group (*p* < 0.05). MET treatment markedly attenuated cleaved caspase-3 immunoreactivity (Figure 8C), resulting in an IRS of 1.62 ± 1.06. AUC treatment produced a further reduction, with IRS values of 0.75 ± 0.71 and 0.38 ± 0.52 in the AUC25 and AUC50 groups, respectively (Figure 8D,E). The IRS values of the MET, AUC25, and AUC50 groups did not differ significantly from those of the NC group.

Expression of Ki67

**Figure 9:**
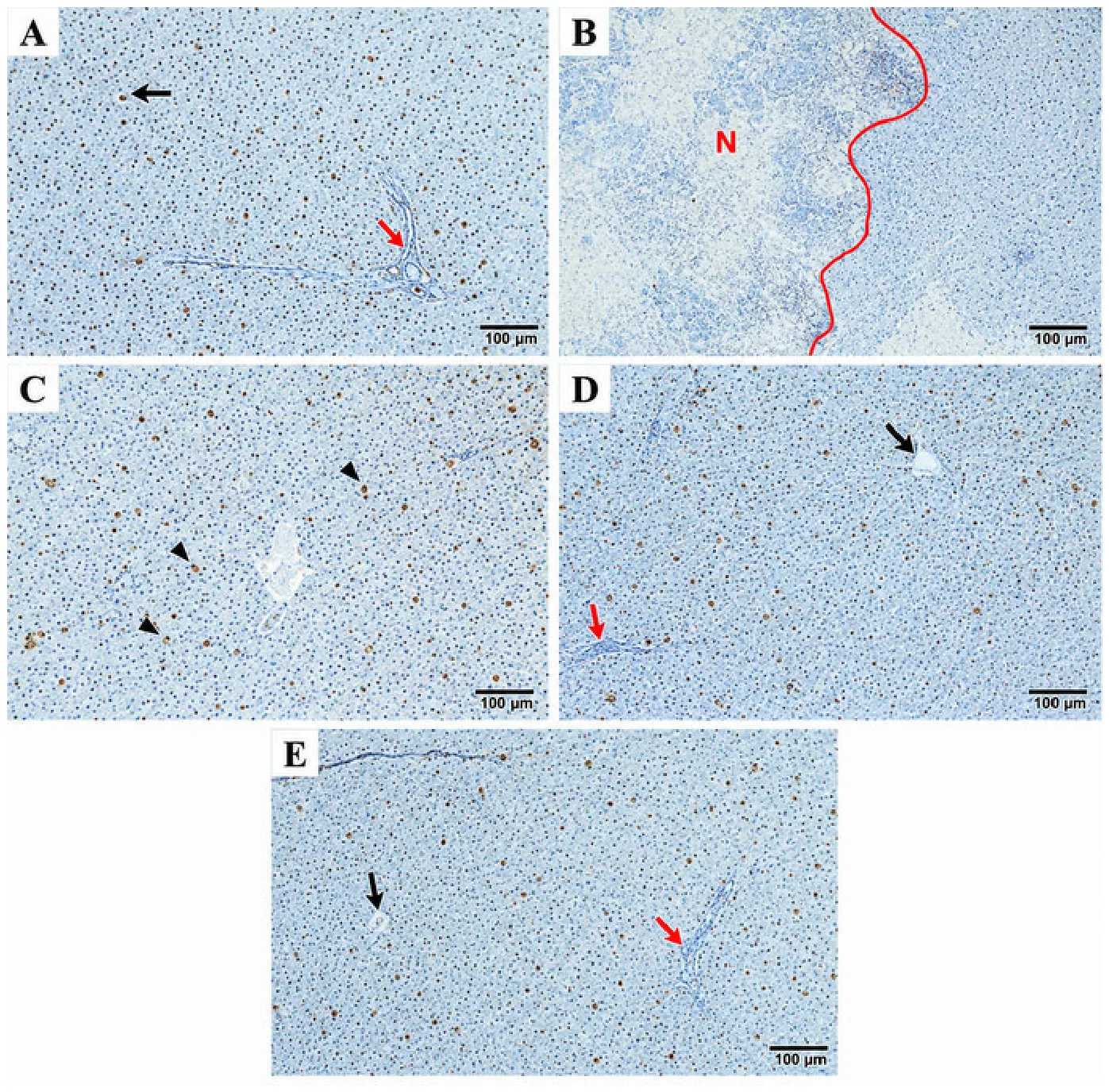
Expression of Ki-67 in hepatic tissue of different study groups: (**A**) Control group showing marked nuclear Ki-67 immunoreactivity in hepatocytes; the central vein (black arrow), portal area (red arrow). (**B**) The alloxan-induced diabetic group did not show Ki-67 expression in either necrotic (N) or viable hepatic tissue; the red line is the border between necrotic and viable hepatic tissue. (**C**) Metformin-treated group showing restoration of Ki-67-positive nuclei in hepatocytes (black arrowheads); the central vein (black arrow). (**D**) Aucubin-treated group at 25 mg/kg (AUC25) demonstrates moderate Ki-67 immunoreactivity in hepatocyte nuclei; the central vein (black arrow), portal area (red arrow). (**E**) Aucubin-treated group at 50 mg/kg (AUC50) had numerous positively stained hepatocyte nuclei throughout the hepatic parenchyma; the central vein (black arrow), portal area (red arrow). Immunostained sections; magnification is ×100 for all.

Ki-67 expression, a marker of cellular proliferation, was observed as brown nuclear immunostaining in hepatocytes. The NC group exhibited prominent nuclear Ki-67 immunoreactivity throughout the hepatic parenchyma (Figure 9A), with a Ki-67 labeling index of 62.00 ± 15.81%. In contrast, the ALX group showed an almost complete absence of Ki-67-positive hepatocyte nuclei in both necrotic and viable areas (Figure 9B), resulting in a markedly reduced labeling index of 0.30 ± 0.32% compared with the NC group (*p* < 0.05). MET treatment partially restored Ki-67 expression, with a moderate number of positive hepatocyte nuclei (Figure 9C) and a labeling index of 41.92 ± 29.70%; however, this value remained significantly lower than that of the NC group. A greater restoration of Ki-67 immunoreactivity was observed following aucubin treatment. The AUC25 group exhibited a moderate number of Ki-67-positive hepatocyte nuclei (Figure 9D), with a labeling index of 60.38 ± 12.86%, whereas the AUC50 group showed numerous positive hepatocyte nuclei distributed throughout the hepatic parenchyma (Figure 9E), with a labeling index of 80.12 ± 11.63%. The Ki-67 labeling indices of the AUC25 and AUC50 groups did not differ significantly from that of the NC group. Notably, aucubin treatment was associated with restoration of hepatic proliferative activity in alloxan-induced diabetic rats, with the 50 mg/kg dose showing the highest Ki-67 labeling index.

## 3. Discussion

Diabetes mellitus is a metabolic disorder characterized by persistent hyperglycemia and profound disturbances in glucose and lipid metabolism. Chronic hyperglycemia promotes excessive reactive oxygen species (ROS) generation, depletion of endogenous antioxidant defenses, inflammatory signaling, mitochondrial dysfunction, and apoptosis, thereby contributing to progressive hepatic injury. The liver is particularly vulnerable to these disturbances because of its central role in glucose and lipid homeostasis. Recent evidence has highlighted the importance of Nrf2-regulated antioxidant responses and NF-κB-associated inflammatory signaling in diabetic complications, supporting the investigation of compounds capable of simultaneously modulating oxidative stress and inflammation [27,28,29].

The present study demonstrates that aucubin attenuated metabolic and hepatic abnormalities in alloxan-induced diabetic rats. The protective effects were reflected by improved body weight and fasting blood glucose, amelioration of serum liver function biomarkers and lipid abnormalities, restoration of hepatic antioxidant status, reduction in inflammatory cytokines, modulation of apoptosis-related proteins, and improvement of hepatic histopathology. Among the tested doses, aucubin at 50 mg/kg generally produced greater effects than the 25 mg/kg dose, indicating a greater overall protective effect at the higher dose under the present experimental conditions.

Alloxan administration produced the expected diabetic phenotype, characterized by persistent hyperglycemia and progressive body-weight loss. Alloxan-induced β-cell injury is primarily associated with ROS generation and subsequent impairment of insulin production, resulting in reduced glucose utilization and increased dependence on lipid and protein catabolism for energy [27,30]. The marked elevation in fasting blood glucose observed in the diabetic group therefore confirms the successful establishment of the experimental diabetic model.

Aucubin significantly reduced fasting blood glucose and improved body weight, particularly at 50 mg/kg. Although the precise mechanism responsible for the antihyperglycemic effect was not directly investigated, improved glycemic control may have contributed to attenuation of the metabolic abnormalities associated with diabetes. Previous studies have also reported antidiabetic and antioxidant effects of aucubin and related phytochemicals [31,32,33]. However, because insulin concentrations and pancreatic β-cell morphology were not investigated in the present study, the contribution of direct pancreatic effects to the observed improvement in glycemic control remains to be established.

Diabetic rats also exhibited significant elevations in AST, ALT, and ALP together with reduced serum albumin, indicating impaired hepatic function. These abnormalities are consistent with diabetes-associated hepatocellular injury, in which oxidative damage, inflammatory responses, and altered cellular membrane integrity can promote leakage of intracellular enzymes into the circulation and impair hepatic synthetic capacity [34,35,36].

Aucubin significantly improved these biochemical parameters, with the 50 mg/kg dose producing values approaching those of the normal control group. These findings suggest that aucubin attenuates diabetes-associated hepatic dysfunction, although the precise contribution of improved glycemic control versus direct hepatic actions cannot be distinguished from the present experimental design.

Diabetes was additionally associated with marked dyslipidemia, including increased total cholesterol, triglycerides, and LDL cholesterol and reduced HDL cholesterol. Insulin deficiency and impaired insulin signaling can increase adipose lipolysis and hepatic fatty-acid delivery while disturbing lipid synthesis and clearance, thereby promoting dyslipidemia [37,38,39,40]. Aucubin significantly improved these lipid abnormalities, particularly at 50 mg/kg. This effect may be related, at least in part, to improved metabolic homeostasis and attenuation of oxidative stress. Previous findings have similarly suggested that aucubin can influence hepatic lipid metabolism and oxidative responses [22].

Oxidative stress represents an important mechanism linking chronic hyperglycemia to hepatic injury. Excessive ROS generation can promote lipid peroxidation, protein and DNA oxidation, mitochondrial dysfunction, and depletion of antioxidant defenses. Consistent with this mechanism, diabetic rats in the present study showed increased hepatic MDA accompanied by depletion of GSH and reductions in SOD and CAT activities [41,42,43].

Aucubin substantially reversed these abnormalities, with the 50 mg/kg dose producing the greatest improvement in hepatic redox status. The restoration of GSH, SOD, and CAT together with reduction in MDA indicates that aucubin strengthens endogenous antioxidant defenses and limits lipid peroxidation. These findings are consistent with previous experimental studies reporting antioxidant properties of aucubin in different models of tissue injury [22,44,45].

The antioxidant response was accompanied by increased hepatic Nrf2 and HO-1 protein expression following aucubin treatment. Nrf2 is an important regulator of cellular antioxidant and cytoprotective responses, whereas HO-1 represents a downstream cytoprotective enzyme [46,47]. The present findings therefore suggest that aucubin may modulate Nrf2/HO-1-associated antioxidant responses in diabetic liver injury. However, because the present Western blot analysis assessed total protein abundance rather than subcellular localization, the observed increase in total Nrf2 expression cannot by itself establish functional Nrf2 activation or nuclear translocation.

Similarly, aucubin reduced total NF-κB p65 protein expression and hepatic TNF-α and IL-6 concentrations. These findings support attenuation of NF-κB p65-associated inflammatory signaling; however, because phosphorylated NF-κB p65 and nuclear translocation were not assessed, definitive inhibition of NF-κB activation cannot be concluded.

Oxidative stress and inflammation are closely interconnected during diabetic hepatic injury. ROS can stimulate NF-κB-associated inflammatory signaling, leading to increased production of cytokines such as TNF-α and IL-6, which in turn can further aggravate oxidative and cellular injury [44,48]. In agreement with this mechanism, untreated diabetic rats exhibited increased hepatic TNF-α and IL-6 levels together with increased NF-κB p65 protein expression.

Aucubin significantly reduced hepatic TNF-α and IL-6 concentrations and attenuated NF-κB p65 protein expression. These findings indicate that aucubin exerts an anti-inflammatory effect in diabetic liver injury and are consistent with previous reports describing anti-inflammatory actions of aucubin in experimental models [48,49,50]. The simultaneous improvement in oxidative stress and inflammatory biomarkers further supports an interaction between redox and inflammatory processes.

Because phosphorylated NF-κB p65 and its nuclear translocation were not assessed, the present data cannot definitively establish inhibition of NF-κB activation. The findings instead indicate attenuation of NF-κB p65-associated inflammatory signaling.

Persistent oxidative and inflammatory stress can promote mitochondrial dysfunction and activate apoptosis. In the present study, diabetes was associated with increased hepatic Bax and reduced Bcl-2 expression, indicating disruption of the balance between pro- and anti-apoptotic signals. Bax promotes mitochondrial outer-membrane permeabilization and facilitates downstream apoptotic signaling, whereas Bcl-2 contributes to preservation of mitochondrial integrity [46,48].

Aucubin treatment reversed these changes, as demonstrated by reduced Bax and increased Bcl-2 expression, particularly at 50 mg/kg. The concomitant reduction in cleaved caspase-3 immunoreactivity further supports attenuation of apoptosis-associated processes. These findings are consistent with previous reports describing the ability of aucubin to modulate Bax/Bcl-2-associated apoptotic signaling in experimental tissue injury [44,50,51].

The anti-apoptotic effect of aucubin may therefore represent an important component of its hepatoprotective activity. Nevertheless, the present findings primarily demonstrate changes in apoptosis-associated proteins and immunoreactivity; they do not independently establish the complete sequence of mitochondrial apoptotic events. Further studies incorporating additional mitochondrial and apoptotic markers could provide more direct mechanistic confirmation.

The biochemical and molecular findings were supported by marked histopathological abnormalities in diabetic rats, including hepatocellular degeneration, cytoplasmic vacuolation, sinusoidal congestion, inflammatory cell infiltration, and focal necrosis. These alterations are consistent with diabetes-associated hepatic injury and corresponded with the observed abnormalities in serum liver enzymes and hepatic oxidative and inflammatory markers.

Aucubin treatment attenuated these pathological changes, with the 50 mg/kg dose showing the greatest preservation of hepatic architecture. The histological improvement was accompanied by reductions in NF-κB and cleaved caspase-3 immunoreactivity and increased Ki-67 immunoreactivity. Collectively, these observations indicate that aucubin treatment was associated with preservation of hepatic tissue integrity and attenuation of inflammatory and apoptosis-related changes.

The increased Ki-67 immunoreactivity indicates enhanced cellular proliferative activity; however, because hepatocyte-specific co-localization was not performed, these findings cannot definitively establish hepatocellular regeneration.

Similarly, the interpretation of Nrf2 immunohistochemistry should be restricted to the staining pattern demonstrated experimentally. In particular, claims of enhanced nuclear Nrf2 should not be made unless nuclear localization is clearly demonstrated. This distinction is important because subcellular localization, rather than total protein abundance alone, provides stronger evidence for functional Nrf2 activation.

Taken together, the findings indicate that aucubin provides multifaceted protection against alloxan-associated diabetic hepatic injury. The observed effects involve improvement of glycemic and lipid abnormalities, restoration of hepatic antioxidant defenses, attenuation of inflammatory cytokine production, modulation of apoptosis-associated proteins, and preservation of hepatic tissue architecture. The greater efficacy generally observed with 50 mg/kg suggests that this dose provides stronger protection under the conditions of the present study.

The coordinated changes in Nrf2/HO-1-related antioxidant signaling, NF-κB p65-associated inflammation, and Bax/Bcl-2-associated apoptosis provide a plausible mechanistic framework for the observed hepatoprotection. This interpretation is consistent with previous studies indicating that aucubin possesses antioxidant and anti-inflammatory properties [44,52,53,54]. However, the present findings should be interpreted as evidence of coordinated modulation of these pathways rather than definitive proof of direct pathway activation or inhibition, particularly for Nrf2 and NF-κB, until functional activation markers are confirmed.

An important strength of the present study is the integration of biochemical, oxidative stress, inflammatory, molecular, histopathological, and immunohistochemical assessments within the same experimental model. This multidimensional approach strengthens the evidence that the hepatoprotective effect of aucubin extends beyond improvement in blood glucose alone. At the same time, the study does not establish whether the observed hepatic benefits are primarily a consequence of improved systemic glycemic control or direct hepatic actions of aucubin.

An additional consideration concerns the pharmacokinetic fate of aucubin following oral administration. Aucubin is an iridoid glycoside and may undergo gastrointestinal and metabolic transformation after ingestion. Therefore, the present study does not establish whether the observed hepatic effects are mediated predominantly by intact aucubin, its metabolites, or a combination of parent compound and metabolites. The observed molecular and biochemical changes should consequently be interpreted as tissue-level associations following aucubin administration rather than direct evidence of hepatic target engagement by the intact compound. Future pharmacokinetic and metabolite-profiling studies are warranted to clarify the bioactive species responsible for the observed effects.

Several limitations should be considered. First, the present study used an alloxan-induced diabetic model representing a type 1-like diabetic condition; therefore, the findings cannot be directly generalized to type 2 diabetes or human diabetes. Second, pancreatic histopathology and circulating insulin concentrations were not evaluated, limiting assessment of the contribution of pancreatic β-cell preservation to the antihyperglycemic effect. Third, additional signaling pathways potentially involved in glucose and hepatic metabolism, including AMPK, PI3K/Akt, and Keap1, were not investigated.

Importantly, total Nrf2 and NF-κB p65 protein expression alone does not conclusively establish pathway activation or inhibition. Future studies should therefore evaluate nuclear Nrf2 accumulation and phosphorylated NF-κB p65 relative to total p65 to provide more direct evidence of pathway activity. In addition, HNF4α/Ki-67 co-staining would be valuable for confirming the hepatocyte identity of proliferating cells. Further investigations should also assess the pharmacokinetic properties, long-term safety, and therapeutic efficacy of aucubin in additional diabetic models and determine its potential interactions with established antidiabetic treatments.

Because nuclear Nrf2 translocation was not directly assessed, the present findings do not establish functional Nrf2 activation. Rather, the observed increase in Nrf2 and HO-1 protein expression suggests involvement of Nrf2/HO-1-associated antioxidant signaling.

Overall, the present findings support aucubin as a promising hepatoprotective candidate in experimental diabetes. Its beneficial effects are associated with improved metabolic status, attenuation of oxidative and inflammatory disturbances, modulation of apoptosis-associated signaling, and preservation of hepatic tissue architecture. Further mechanistic and translational studies are required to establish the precise molecular targets responsible for these effects and to determine the therapeutic relevance of aucubin in diabetes-associated liver injury.

## 4. Materials and Methods

### 4.1. Materials and Drugs

Alloxan monohydrate, aucubin, and metformin hydrochloride were obtained from Sigma-Aldrich Co. (St. Louis, MO, USA). Unless otherwise specified, all chemicals and reagents used throughout the study were of analytical grade.

### 4.2. Experimental Animals

Adult male Wistar rats (180–200 g) were obtained from the Animal House, Faculty of Science, Sohag University, Egypt. Animals were housed under standard laboratory conditions (22 ± 2 °C, relative humidity 50–60%, and a 12 h light/12 h dark cycle) with free access to a standard laboratory diet and water throughout the experimental period. All experimental procedures were conducted in accordance with the guidelines approved by the Sohag Institutional Animal Care and Use Committee (Sohag-IACUC), Faculty of Science, Sohag University (Protocol No. SU-FS-26-26).

### 4.3. Induction of Experimental Diabetes Mellitus

Following one week of acclimatization, experimental diabetes mellitus was induced by a single intraperitoneal injection of alloxan monohydrate (150 mg/kg) freshly dissolved in normal saline after an overnight fast. To minimize mortality resulting from alloxan-induced acute hypoglycemia, rats received 5% glucose solution for 24 h following alloxan administration. Seventy-two hours later, fasting blood glucose (FBG) levels were measured using a glucometer, and rats with FBG ≥250 mg/dL were considered diabetic and included in the study.

### 4.4. Experimental Design

To establish a therapeutic rather than a preventive model, diabetic rats were left untreated for 7 days after diabetes confirmation before initiating treatment. Subsequently, animals were randomly allocated into five experimental groups (*n* = 8 per group), and treatments were administered once daily by oral gavage for 28 consecutive days as follows:

Group I (Control): Normal rats receiving the vehicle only.

Group II (ALX): Alloxan-induced diabetic rats receiving the vehicle.

Group III (ALX + MET): Alloxan-induced diabetic rats treated with metformin (150 mg/kg/day, p.o.).

Group IV (ALX + AUC25): Alloxan-induced diabetic rats treated with aucubin (25 mg/kg/day, p.o.).

Group V (ALX + AUC50): Alloxan-induced diabetic rats treated with aucubin (50 mg/kg/day, p.o.).

**Figure 10:**
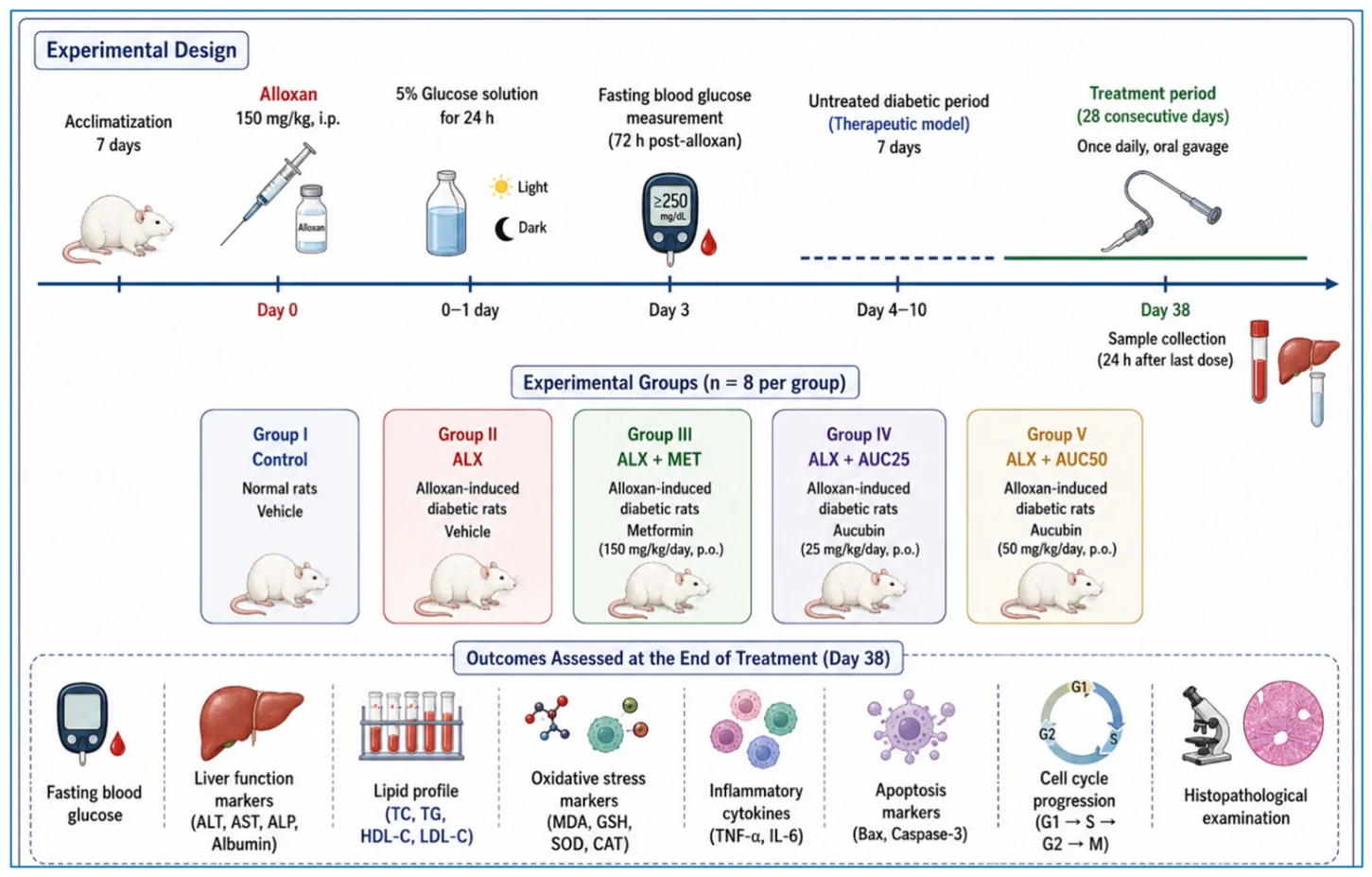
Experimental timeline and study design of the therapeutic protocol for alloxan-induced diabetic liver injury in rats. Following diabetes confirmation (FBG ≥ 250 mg/dL), rats remained untreated for 7 days before receiving oral metformin (150 mg/kg/day) or aucubin (25 or 50 mg/kg/day) for 28 consecutive days. Body weight and FBG were monitored, followed by collection of blood and liver samples for biochemical, molecular, histopathological, and immunohistochemical analyses.

The selected dose of metformin (150 mg/kg/day) was based on previously published experimental studies demonstrating its efficacy as a standard antidiabetic drug in alloxan-induced diabetic rats [55]. Likewise, the doses of aucubin (25 and 50 mg/kg/day) were selected according to previous reports demonstrating significant antioxidant, anti-inflammatory, anti-apoptotic, and hepatoprotective activities in experimental animal models [56,57]. The overall experimental protocol is illustrated in Figure 10.

The vehicle used for oral administration was administered to the control and diabetic control groups at the same volume and under the same administration conditions as the corresponding treatment formulations. Following confirmation of diabetes, animals meeting the predefined inclusion criterion (FBG ≥ 250 mg/dL) were eligible for random allocation to the experimental groups. Randomization was performed using a simple random allocation procedure to minimize allocation bias. The sample size of eight animals per group was selected based on the expected biological variability of the investigated parameters and previous experimental studies using comparable animal models, while adhering to the principle of using the minimum number of animals necessary to obtain reliable results.

Following alloxan administration, animals were monitored for general health and survival. Animals that died following alloxan administration or did not meet the predefined criterion for successful diabetes induction were excluded from the subsequent experimental groups and analyses. The final number of animals analyzed in each group was *n* = 8.

### 4.5. Sample Collection

At the end of the treatment period, animals were fasted overnight and anesthetized before blood samples were collected for biochemical analyses. The liver was immediately excised, rinsed with ice-cold normal saline, blotted dry, and divided into separate portions for biochemical assays, Western blot analysis, histopathological examination, and immunohistochemical evaluation.

### 4.6. Body Weight and Fasting Blood Glucose Assessment

Body weight and fasting blood glucose (FBG) levels were determined at three predefined time points: before diabetes induction (baseline), 72 h after alloxan injection to confirm diabetes induction, and at the end of the 28-day treatment period before animal sacrifice. Body weight was measured using a calibrated digital balance, whereas fasting blood glucose was determined from tail vein blood samples using a portable glucometer after an overnight fast. Rats with fasting blood glucose levels ≥ 250 mg/dL, measured 72 h after alloxan administration, were considered diabetic and enrolled in the study.

### 4.7. Assessment of Liver Function Biomarkers

At the end of the experimental period, blood samples were collected by cardiac puncture under anesthesia and allowed to clot at room temperature. Serum was separated by centrifugation at 3000 rpm for 15 min and stored at −80 °C until analysis.

Serum alanine aminotransferase (ALT), aspartate aminotransferase (AST), alkaline phosphatase (ALP), and albumin levels were determined using commercially available colorimetric diagnostic kits (Bio-diagnostic Co., Giza, Egypt) according to the manufacturer’s instructions. The results were expressed as U/L for ALT, AST, and ALP, and g/dL for albumin.

### 4.8. Assessment of Serum Lipid Profile

Serum total cholesterol (TC), triglycerides (TG), and high-density lipoprotein cholesterol (HDL-C) were determined using commercially available colorimetric diagnostic kits (Bio-diagnostic Co., Giza, Egypt) according to the manufacturer’s instructions. Low-density lipoprotein cholesterol (LDL-C) was calculated using the Friedewald equation: LDL-C = TC − HDL-C − (TG/5), provided that serum TG concentrations were below 400 mg/dL. Serum lipid concentrations were expressed as mg/dL.

### 4.9. Assessment of Hepatic Oxidative Stress Biomarkers

Liver tissues were immediately excised, rinsed with ice-cold physiological saline to remove residual blood, blotted dry, weighed, and homogenized (10% *w*/*v*) in ice-cold phosphate-buffered saline (PBS, pH 7.4). The homogenates were centrifuged at 10,000× *g* for 15 min at 4 °C, and the resulting supernatants were collected and stored at −80 °C until biochemical analysis.

Hepatic oxidative stress status was evaluated by measuring malondialdehyde (MDA), reduced glutathione (GSH), superoxide dismutase (SOD), and catalase (CAT) using commercially available colorimetric assay kits purchased from Nanjing Jiancheng Bioengineering Institute (Nanjing, China) (MDA, Cat. No. A003-1; GSH, Cat. No. A006-2-1; SOD, Cat. No. A001-3; and CAT, Cat. No. A007-1-1), according to the manufacturer’s instructions.

### 4.10. Determination of Hepatic Inflammatory Cytokines

Hepatic concentrations of tumor necrosis factor-α (TNF-α) and interleukin-6 (IL-6) were quantified in liver tissue homogenates. Briefly, liver samples were homogenized in ice-cold phosphate-buffered saline (PBS, pH 7.4), and the homogenates were centrifuged at 10,000× *g* for 15 min at 4 °C. The collected supernatants were used for cytokine determination.

TNF-α and IL-6 concentrations were measured using commercially available rat enzyme-linked immunosorbent assay (ELISA) kits purchased from Elabscience Biotechnology Inc. (Houston, TX, USA), according to the manufacturer’s instructions. TNF-α levels were measured using the Rat TNF-α ELISA Kit (Cat. No. E-EL-R2856), while IL-6 levels were measured using the Rat IL-6 ELISA Kit (Cat. No. E-EL-R0015). Absorbance was recorded using a microplate reader at the wavelength recommended by the manufacturer.

The total protein content of each liver homogenate was determined using the bicinchoninic acid (BCA) protein assay, and cytokine concentrations were normalized to the corresponding total protein content. Cytokine levels were therefore expressed as pg/mg total protein.

### 4.11. Western Blot Analysis

To investigate the molecular mechanisms underlying the therapeutic effects of aucubin on alloxan-induced hepatic injury, the protein expression levels of oxidative stress-, inflammation-, and apoptosis-related markers were evaluated by Western blot analysis.

Frozen liver tissues were homogenized in ice-cold RIPA lysis buffer supplemented with protease and phosphatase inhibitor cocktails. The homogenates were centrifuged at 12,000× *g* for 20 min at 4 °C, and the supernatants containing total cellular proteins were collected. Protein concentrations were determined using the bicinchoninic acid (BCA) protein assay.

Equal amounts of protein (30–50 μg) were separated by 10–12% sodium dodecyl sulfate-polyacrylamide gel electrophoresis (SDS-PAGE) and subsequently transferred onto polyvinylidene difluoride (PVDF) membranes. The membranes were blocked with 5% non-fat dry milk in Tris-buffered saline containing 0.1% Tween-20 (TBST) for 1 h at room temperature and then incubated overnight at 4 °C with primary antibodies against nuclear factor erythroid 2-related factor 2 (Nrf2), heme oxygenase-1 (HO-1), nuclear factor-kappa B p65 (NF-κB p65), B-cell lymphoma-2 (Bcl-2), and Bcl-2-associated X protein (Bax).

Following incubation, the membranes were washed with TBST and incubated with the appropriate horseradish peroxidase (HRP)-conjugated secondary antibodies for 1 h at room temperature. Protein bands were visualized using an enhanced chemiluminescence (ECL) detection system and captured using a gel documentation system. Densitometric analysis was performed using ImageJ software version 1.54 (National Institutes of Health, Bethesda, MD, USA). The expression levels of the target proteins were normalized to β-actin, which served as the internal loading control, and expressed as the relative fold change compared with the control group.

Primary antibodies against Nrf2 (ab62352), HO-1 (ab13243), NF-κB p65 (ab32536), Bax (ab32503), Bcl-2 (ab32124), and β-actin (ab8227), together with horseradish peroxidase (HRP)-conjugated goat anti-rabbit IgG (ab205718) and goat anti-mouse IgG (ab205719) secondary antibodies, were purchased from Abcam (Cambridge, UK).

### 4.12. Histopathological Examination

Liver specimens were immediately excised, rinsed with ice-cold normal saline to remove residual blood, and fixed in 10% neutral-buffered formalin for 24–48 h. The fixed tissues were dehydrated through a graded ethanol series, cleared in xylene, embedded in paraffin wax, and sectioned at a thickness of 4–5 μm using a rotary microtome.

The paraffin sections were stained with hematoxylin and eosin (H&E) according to standard histological procedures and examined under a light microscope by a blinded histopathologist. Histopathological evaluation was performed to assess hepatic structural alterations, including hepatocellular degeneration, cytoplasmic vacuolization, hepatocyte necrosis, inflammatory cell infiltration, sinusoidal dilatation, vascular congestion, and disruption of the normal hepatic architecture.

Histopathological changes, including hepatocellular degeneration (Cloudy swelling/Vacuolar Degeneration), necrosis, inflammatory cell infiltration, congestion and/or dilatation, and steatosis, were scored according to their severity (− = none; + = mild; ++ = moderate; +++ = severe). Individual lesion scores were summed to generate a total histopathological injury score ranging from 0 to 15. The overall severity of hepatic injury was categorized as normal (0–1), mild (2–4), moderate (5–9), or severe (10–15), as detailed in Table 6.

### 4.13. Immunohistochemical Analysis

Paraffin-embedded liver tissues were sectioned at a thickness of 4 μm and mounted on positively charged glass slides. The sections were deparaffinized in xylene and rehydrated through graded ethanol to distilled water. Heat-induced antigen retrieval was performed by heating the sections in 10 mM sodium citrate buffer (pH 6.0) in a microwave oven for 15 min. Endogenous peroxidase activity was quenched with 0.3% hydrogen peroxide (H_2_O_2_) for 15 min at room temperature. Following three washes with phosphate-buffered saline (PBS), nonspecific binding sites were blocked with normal goat serum for 15 min.

The sections were incubated overnight at 4 °C with the following primary antibodies: rabbit anti-cleaved caspase-3 polyclonal antibody (GB11532, 1:1000; Servicebio, Wuhan, China), rabbit anti-nuclear factor erythroid 2-related factor 2 (Nrf2) polyclonal antibody (GB113808, 1:1000; Servicebio, Wuhan, China), rabbit anti-Ki-67 monoclonal antibody (clone SP6, RM0255, 1:100; Medaysis, Livermore, CA, USA), and mouse anti-NF-κB p65 monoclonal antibody (clone G-8, sc-398442, 1:50; Santa Cruz Biotechnology, Dallas, TX, USA).

After washing with PBS, the sections were incubated with the secondary antibody, which was the Histofine simple stain MAX-PO (Nichirei Bioscience, Inc., Tokyo, Japan). The 3,3-diaminobenzidine substrate (Millipore Sigma, Tokyo, Japan) and hematoxylin were used to visualize IHC stains.

Immunohistochemical expression of NF-κB (p65), Nrf2, cleaved caspase-3, and Ki-67 was evaluated in liver sections from all experimental groups. NF-κB (p65), Nrf2, and cleaved caspase-3 immunoreactivity was assessed semi-quantitatively according to the proportion of immunopositive hepatocytes and staining intensity. The percentage of positive cells was scored as 0 = 0%, 1 = 1–25%, 2 = 26–50%, 3 = 51–75%, and 4 = >75%. Staining intensity was scored as 0 = absent, 1 = weak, 2 = moderate, and 3 = intense. The immunoreactivity score (IRS) was calculated by multiplying the percentage-positive cell score by the staining-intensity score, with a maximum possible score of 12 [58]. The Ki-67 labeling index was calculated as the percentage of Ki-67-positive hepatocyte nuclei among the total hepatocyte nuclei examined. Eight animals were evaluated per experimental group.

### 4.14. Statistical Analysis

All data were expressed as mean ± standard deviation (SD) for eight animals per group (*n* = 8). Statistical analyses were performed using IBM SPSS Statistics software (Version 27.0; IBM Corp., Armonk, NY, USA). Data normality was assessed using the Shapiro–Wilk test, and homogeneity of variances was evaluated using Levene’s test.

Comparisons among multiple experimental groups were performed using one-way analysis of variance (ANOVA) followed by Tukey’s post hoc multiple comparison test. A value of *p* < 0.05 was considered statistically significant.

For body weight and fasting blood glucose, which were measured repeatedly in the same animals at different time points, a repeated-measures ANOVA was performed, with time (Day 0, Day 3, and Day 38) considered as the within-subject factor and experimental group as the between-subject factor. The group × time interaction was also evaluated to determine whether the temporal changes differed among the experimental groups. Tukey’s multiple comparison test was used for post hoc comparisons where appropriate. Statistical significance was considered at *p* < 0.05.

For the semi-quantitative immunohistochemical data, including the immunoreactivity scores (IRS) for NF-κB (p65), Nrf2, and cleaved caspase-3, and the Ki-67 labeling index, the Kruskal–Wallis test followed by Dunn’s multiple-comparison test was used. A value of *p* < 0.05 was considered statistically significant.

## 5. Conclusions

In conclusion, the present study demonstrates that aucubin exerts hepatoprotective effects against alloxan-induced diabetic liver injury, as reflected by improvements in oxidative stress, inflammation, apoptosis, and hepatic architecture. These beneficial effects were associated with enhanced Nrf2/HO-1 signaling, attenuation of NF-κB p65 expression and pro-inflammatory cytokines, modulation of the Bax/Bcl-2 balance, and increased Ki-67 immunoreactivity, suggesting enhanced cellular proliferative activity. These molecular and cellular effects were accompanied by significant improvements in glycemic status, body weight, liver function, lipid homeostasis, and hepatic antioxidant status, together with amelioration of histopathological alterations. Notably, the 50 mg/kg dose generally produced greater effects than the 25 mg/kg dose across the evaluated parameters, supporting its greater efficacy under the present experimental conditions.

To the best of our knowledge, the present study provides further evidence that aucubin ameliorates alloxan-induced diabetic hepatic injury, as reflected by improvements in hepatic function, lipid metabolism, oxidative stress, inflammation, and apoptosis. These protective effects were associated with enhanced Nrf2/HO-1 signaling, attenuation of NF-κB p65 expression and pro-inflammatory cytokines, and modulation of the Bax/Bcl-2 balance. Increased Ki-67 immunoreactivity further suggested enhanced cellular proliferative activity, although this finding alone does not establish hepatocellular regeneration. Collectively, these findings support further investigation of aucubin as a potential hepatoprotective agent in diabetes-associated liver injury. Further pharmacokinetic, toxicological, and clinical studies are warranted to clarify its long-term efficacy, safety, and translational potential.

## Figures and Tables

**Table 1:** Effect of aucubin on serum liver function biomarkers in alloxan-induced diabetic rats.

Groups/Parameters	AST (U/L)	ALT (U/L)	ALP (U/L)	Albumin (g/dL)
**NC**	65.01 ± 2.35	43.94 ± 2.16	53.70 ± 4.13	5.29 ± 0.50
**ALX**	190.64 ± 4.39 ***	176.55 ± 4.04 ***	114.12 ± 4.19 ***	2.10 ± 0.11 ***
**ALX + MET**	115.07 ± 4.23 **^,##^	97.86 ± 4.89 **^,##^	63.45 ± 1.94 ^ns,###^	3.10 ± 0.09 ***^,##^
**ALX + AUC25**	81.39 ± 3.35 *^,###^	60.46 ± 3.77 *^,###^	71.86 ± 1.95 *^,###^	3.56 ± 0.223 **^,###^
**ALX + AUC50**	77.33 ± 2.66 ^ns,###^	49.44 ± 2.36 ^ns,###^	62.37 ± 2.55 ^ns,###^	4.03 ± 0.12 ^ns,###^

Data are presented as mean ± SD (*n* = 8 rats per group). Statistical analysis was performed using one-way ANOVA followed by Tukey’s multiple comparison test. ns, not significant; * *p* < 0.05, ** *p* < 0.01, and *** *p* < 0.001 versus the normal control (NC) group; ^##^ *p* < 0.01, and ^###^ *p* < 0.001 versus the alloxan-induced diabetic (ALX) group. NC, normal control; ALX, alloxan-induced diabetic group; MET, metformin (reference antidiabetic drug); AUC25, aucubin (25 mg/kg); AUC50, aucubin (50 mg/kg); AST, aspartate aminotransferase; ALT, alanine aminotransferase; ALP, alkaline phosphatase.

**Table 2:** Effect of aucubin on serum lipid profile in alloxan-induced diabetic rats.

Groups/Parameters	CH (mg/dL)	TG (mg/dL)	HDL (mg/dL)	LDL (mg/dL)
**NC**	80.82 ± 1.52	51.09 ± 1.48	29.20 ± 2.47	41.40 ± 2.28
**ALX**	185.60 ± 2.39 ***	148.29 ± 2.36 ***	4.08 ± 1.27 ***	151.86 ± 1.95 ***
**ALX + MET**	113.41 ± 2.78 **^,##^	96.42 ± 3.07 **^,##^	21.08 ± 1.31 **^,##^	73.03 ± 2.12 **^,##^
**ALX + AUC25**	100.85 ± 1.99 *^,##^	82.39 ± 1.92 *^,##^	22.95 ± 1.77 *^,##^	61.42 ± 2.65 *^,##^
**ALX + AUC50**	94.26 ± 2.67 ^ns,###^	69.55 ± 2.31 ^ns,###^	23.79 ± 1.48 ^ns,###^	56.56 ± 3.50 ^ns,###^

Data are presented as mean ± SD (*n* = 8 rats per group). Statistical analysis was performed using one-way ANOVA followed by Tukey’s multiple comparison test. ns, not significant; * *p* < 0.05, ** *p* < 0.01, and *** *p* < 0.001 versus the normal control (NC) group; ^##^ *p* < 0.01, and ^###^ *p* < 0.001 versus the alloxan-induced diabetic (ALX) group. NC, normal control; ALX, alloxan-induced diabetic group; MET, metformin (reference antidiabetic drug); AUC25, aucubin (25 mg/kg); AUC50, aucubin (50 mg/kg); CH, total cholesterol; TG, triglycerides; HDL, high-density lipoprotein cholesterol; LDL, low-density lipoprotein cholesterol.

**Table 3:** Effect of aucubin on hepatic oxidative stress biomarkers in alloxan-induced diabetic rats.

Groups/Parameters	MDA (nmol/g Tissue)	GSH (mg/g Tissue)	SOD (U/mg Protein)	CAT U/g Tissue
**NC**	9.33 ± 1.21	49.23 ± 1.563	28.15 ± 1.80	3.16 ± 0.37
**ALX**	31.80 ± 1.41 ***	18.53 ± 1.36 ***	9.29 ± 1.10 ***	1.69 ± 0.29 ***
**ALX + MET**	24.88 ± 1.39 **^,##^	24.36 ± 1.71 **^,##^	14.34 ± 1.63 **^,##^	2.00 ± 0.09 **^,##^
**ALX + AUC25**	19.06 ± 1.43 *^,###^	34.82 ± 1.37 *^,###^	19.87 ± 1.82 *^,###^	2.32 ± 0.22 *^,###^
**ALX + AUC50**	12.66 ± 0.705 ^ns,###^	41.99 ± 1.60 ^ns,###^	22.07 ± 2.087 ^ns,###^	2.87 ± 0.31 ^ns,###^

Data are presented as mean ± SD (*n* = 8 rats per group). Statistical analysis was performed using one-way ANOVA followed by Tukey’s multiple comparison test. ns, not significant; * *p* < 0.05, ** *p* < 0.01, and *** *p* < 0.001 versus the normal control (NC) group; ^##^ *p* < 0.01, and ^###^ *p* < 0.001 versus the alloxan-induced diabetic (ALX) group. NC, normal control; ALX, alloxan-induced diabetic group; MET, metformin (reference antidiabetic drug); AUC25, aucubin (25 mg/kg); AUC50, aucubin (50 mg/kg); MDA, malondialdehyde; GSH, reduced glutathione; SOD, superoxide dismutase; CAT, catalase.

**Table 4:** Histopathologic findings in the liver tissues of each experimental group.

Finding/ Group	Cloudy Swelling/Vacuolar Degen	Necrosis	Inflammation	Congestion/Dilatation	Steatosis	Severity of the Injury
**NC**	−	−	−	−	−	Normal
**ALX**	+++	+++	++	++	+++	Severe
**ALX + MET**	++	++	+	+	+	Moderate
**ALX + AUC25**	+	−	+	+	−	Mild
**ALX + AUC50**	+	−	−	+	−	Mild

Scoring:− = normal; + = mild; ++ = moderate; +++ = severe.

**Table 5:** Semi-quantitative immunohistochemical evaluation of hepatic NF-κB (p65), Nrf2, cleaved caspase-3, and Ki-67 expression in each experimental group.

Experimental Group	NF-κB (p65) IRS	Nrf2 IRS	Cleaved Caspase-3 IRS	Ki-67 Labeling Index (%)
**NC**	0.50 ± 0.76 ᵃ	6.50 ± 2.00 ᵃ	0.62 ± 0.92 ᵃ	62.00 ± 15.81 ᵃ
**ALX**	8.50 ± 2.51 ᵇ	0.50 ± 0.76 ᵇ	9.75 ± 2.66 ᵇ	0.30 ± 0.32 ᵇ
**MET**	2.00 ± 1.07 ᵃ	2.50 ± 1.31 ᵇ	1.62 ± 1.06 ᵃ	41.92 ± 29.70 ᵇ
**AUC25**	0.62 ± 0.74 ᵃ	4.38 ± 2.13 ᵃ	0.75 ± 0.71 ᵃ	60.38 ± 12.86 ᵃ
**AUC50**	0.38 ± 0.52 ᵃ	6.38 ± 1.85 ᵃ	0.38 ± 0.52 ᵃ	80.12 ± 11.63 ᵃ

Data are presented as mean ± SD (*n* = 8 rats per group). IRS, immunoreactivity score. IRS was calculated by multiplying the percentage-positive cell score by the staining-intensity score. Percentage-positive cells were scored as 0 = 0%, 1 = 1–25%, 2 = 26–50%, 3 = 51–75%, and 4 = >75%, while staining intensity was scored as 0 = absent, 1 = weak, 2 = moderate, and 3 = intense, giving a maximum IRS of 12. The Ki-67 labeling index was calculated as the percentage of Ki-67-positive hepatocyte nuclei among the total hepatocyte nuclei examined. Statistical analysis was performed using the Kruskal–Wallis test followed by Dunn’s multiple-comparison test. Different superscript lowercase letters within the same column indicate statistically significant differences among groups (*p* < 0.05), whereas groups sharing the same superscript letter are not significantly different. NC, normal control; ALX, alloxan-induced diabetic group; MET, metformin (reference antidiabetic drug); AUC25, aucubin (25 mg/kg); AUC50, aucubin (50 mg/kg).

**Table 6:** Criteria used for the histopathologic scoring of the liver tissues of each experimental group.

Histopathological Features	Points
**A.** Hepatocellular degeneration (cloudy swelling/vacuolar degeneration)	
− = Absent	0
+ = Mild	1
++ = Moderate	2
+++ = Severe	3
**B.** Necrosis	
− = Absent	0
+ = Focal necrosis	1
++ = Multifocal necrosis	2
+++ = Extensive/geographical necrosis	3
**C.** Congestion and/or dilatation	
− = absent	0
+ = Mild	1
++ = Moderate	2
+++ = Severe	3
**D.** Inflammation	
− = Absent	0
+ = Mild/focal	1
++ = Moderate/multifocal	2
+++ = Severe/diffuse	3
**E.** Steatosis	
− = Absent	0
+ = <25% hepatocytes	1
++ = 25–50%	2
+++ = >50%	3
Total score: A + B + C + D + E	Total
Normal	0–1
Mild injury	2–4
Moderate injury	5–9
Severe injury	10–15

Note: Each histopathological feature was scored on a 0–3 scale, where 0 indicates absence, 1 indicates mild or focal changes, 2 indicates moderate or multifocal changes, and 3 indicates severe, diffuse, extensive, or geographical changes, according to the specific lesion. The total histopathological injury score was calculated as the sum of scores for hepatocellular degeneration (A), necrosis (B), congestion and/or dilatation (C), inflammation (D), and steatosis (E), yielding a maximum possible score of 15. The total score was interpreted as follows: 0–1, normal; 2–4, mild injury; 5–9, moderate injury; and 10–15, severe injury.

## Data Availability

All data generated or analyzed during this study are included in this published article.

## Abbreviations

ALP	Alkaline phosphatase
ALX	Alloxan-induced diabetic group
ALT	Alanine aminotransferase
AST	Aspartate aminotransferase
AUC25	Aucubin (25 mg/kg)
AUC50	Aucubin (50 mg/kg)
Bax	Bcl-2-associated X protein
Bcl-2	B-cell lymphoma 2
CAT	Catalase
CH	Total cholesterol
DM	Diabetes mellitus
GSH	Reduced glutathione
HDL	High-density lipoprotein cholesterol
HO-1	Heme oxygenase-1
IL-6	Interleukin-6
LDL	Low-density lipoprotein cholesterol
MDA	Malondialdehyde
MET	Metformin
NC	Normal control
NF-κB	Nuclear factor kappa B
Nrf2	Nuclear factor erythroid 2-related factor 2
SOD	Superoxide dismutase
TG	Triglycerides
TNF-α	Tumor necrosis factor-alpha
